# Mapping single-cell data to reference atlases by transfer learning

**DOI:** 10.1038/s41587-021-01001-7

**Published:** 2021-08-30

**Authors:** Mohammad Lotfollahi, Mohsen Naghipourfar, Malte D. Luecken, Matin Khajavi, Maren Büttner, Marco Wagenstetter, Žiga Avsec, Adam Gayoso, Nir Yosef, Marta Interlandi, Sergei Rybakov, Alexander V. Misharin, Fabian J. Theis

**Affiliations:** 1 Helmholtz Center Munich—German Research Center for Environmental Health, Institute of Computational Biology, Neuherberg, Germany; 2 School of Life Sciences Weihenstephan, Technical University of Munich, Munich, Germany; 3 Department of Computer Science, Technical University of Munich, Munich, Germany; 4 Center for Computational Biology, University of California, Berkeley, Berkeley, CA USA; 5 Department of Electrical Engineering and Computer Sciences, University of California, Berkeley, Berkeley, CA USA; 6 Chan Zuckerberg Biohub, San Francisco, CA USA; 7 Ragon Institute of MGH, MIT and Harvard, Cambridge, MA USA; 8 Institute of Medical Informatics, University of Münster, Münster, Germany; 9 Department of Mathematics, Technical University of Munich, Munich, Germany; 10 Division of Pulmonary and Critical Care Medicine, Feinberg School of Medicine, Northwestern University, Chicago, IL USA

**Keywords:** Machine learning, Data integration

## Abstract

Large single-cell atlases are now routinely generated to serve as references for analysis of smaller-scale studies. Yet learning from reference data is complicated by batch effects between datasets, limited availability of computational resources and sharing restrictions on raw data. Here we introduce a deep learning strategy for mapping query datasets on top of a reference called single-cell architectural surgery (scArches). scArches uses transfer learning and parameter optimization to enable efficient, decentralized, iterative reference building and contextualization of new datasets with existing references without sharing raw data. Using examples from mouse brain, pancreas, immune and whole-organism atlases, we show that scArches preserves biological state information while removing batch effects, despite using four orders of magnitude fewer parameters than de novo integration. scArches generalizes to multimodal reference mapping, allowing imputation of missing modalities. Finally, scArches retains coronavirus disease 2019 (COVID-19) disease variation when mapping to a healthy reference, enabling the discovery of disease-specific cell states. scArches will facilitate collaborative projects by enabling iterative construction, updating, sharing and efficient use of reference atlases.

## Abstract (web summary)

Single-cell data are readily integrated with cell atlases using scArches.

## Main

Large single-cell reference atlases^1–4^ comprising millions^5^ of cells across tissues, organs, developmental stages and conditions are now routinely generated by consortia such as the Human Cell Atlas^6^. These references help to understand the cellular heterogeneity that constitutes natural and inter-individual variation, aging, environmental influences and disease. Reference atlases provide an opportunity to radically change how we currently analyze single-cell datasets: by learning from the appropriate reference, we could automate annotation of new datasets and easily perform comparative analyses across tissues, species and disease conditions.

Learning from a reference atlas requires mapping a query dataset to this reference to generate a joint embedding. Yet query datasets and reference atlases typically comprise data generated in different laboratories with different experimental protocols and thus contain batch effects. Data-integration methods are typically used to overcome these batch effects in reference construction^7^. This requires access to all relevant datasets, which can be hindered by legal restrictions on data sharing. Furthermore, contextualizing a single dataset requires rerunning the full integration pipeline, presupposing both computational expertise and resources. Finally, traditional data-integration methods consider any perturbation between datasets that affects most cells as a technical batch effect, but biological perturbations may also affect most cells. Thus, conventional approaches are insufficient for mapping query data onto references across biological conditions.

Exploiting large reference datasets is a well-established approach in Computer Vision^8^ and Natural Language Processing^9^. In these fields, commonly used deep learning approaches typically require a large number of training samples, which are not always available. By leveraging weights learned from large reference datasets to enhance learning on a target or query dataset^10^, transfer-learning (TL) models such as ImageNet^11^ and BERT^12^ have revolutionized analysis approaches^8,9^: TL has improved method performance with small datasets (for example, clustering^13^, classification and/or annotation^14^) and enabled model sharing^15–18^. Recently, TL has been applied to single-cell RNA-seq (scRNA-seq) data for denoising^19^, variance decomposition^20^ and cell type classification^21,22^. However, current TL approaches in genomics do not account for technical effects within and between the reference and query^19^ and lack of systematic retraining with query data^20–23^. These limitations can lead to spurious predictions on query data with no or small overlap in cell types, tissues or species^24,25^. Nonetheless, deep learning models for data integration in single-cell genomics demonstrated superior performance^7,26–28^. We propose a TL and fine-tuning strategy to leverage existing conditional neural network models and transfer them to new datasets, called ‘architecture surgery’, as implemented in the scArches pipeline. scArches is a fast and scalable tool for updating, sharing and using reference atlases trained with a variety of neural network models. Specifically, given a basic reference atlas, scArches enables users to share this reference as a trained network with other users, who can in turn update the reference using query-to-reference mapping and partial weight optimization without sharing their data. Thus, users can build their own extended reference models or perform stepwise analysis of datasets as they are collected, which is often crucial for emerging clinical datasets. Furthermore, scArches allows users to learn from reference data by contextualizing new (for example, disease) data with a healthy reference in a shared representation. Due to the flexible choice of the underlying core model that is transferred using scArches, we can learn references with various base models but also train on multimodal data. We demonstrate the features of scArches using single-cell datasets ranging from pancreas to whole-mouse atlases and immune cells from patients with COVID-19. scArches is able to iteratively update a pancreas reference, transfer labels or unmeasured data modalities between reference atlases and query data and map COVID-19 data onto a healthy reference while preserving disease-specific variation.

## Results

### scArches enables mapping query data to reference

**Fig. 1:**
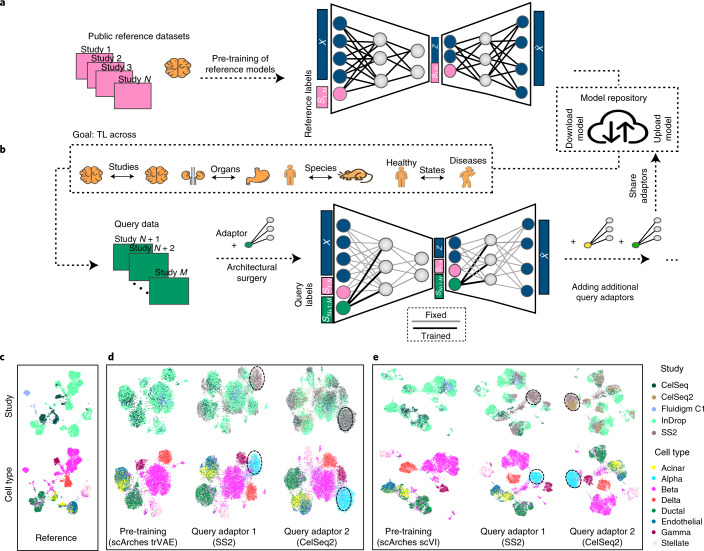
scArches enables iterative query-to-reference single-cell integration. **a**, Pre-training of a latent representation using public reference datasets and corresponding reference labels. **b**, Decentralized model building: users download parameters for the atlas of interest, fine tune the model and optionally upload their updated model for other users. **c**–**e**, Illustration of this workflow for a human pancreas atlas across different scArches base models. Training a reference atlas across three human pancreas datasets (CelSeq, InDrop, Fluidigm C1), uniform manifold approximation and projection (UMAP) embedding for the original (**c**) and the integrated reference for pre-trained reference models (**d**,**e**, first column). Second column in **d**,**e**, querying a new SS2 dataset to the integrated reference. Updating the cell atlas with a fifth dataset (CelSeq2). Third column in **d**,**e**, black dashed circles represent cells absent in the reference data. UMAP plots are based on the model embedding.

Consider the scenario with *N* ‘reference’ scRNA-seq datasets of a particular tissue or organism. A common approach to integrate such datasets is to use a conditional variational autoencoder (CVAE) (for example, single-cell variational inference (scVI)^29^, transfer variational autoencoder (trVAE)^30^) that assigns a categorical label *S*_*i*_ to each dataset that corresponds to the study label. These study labels may index traditional batch IDs (that is, samples, experiments across laboratories or sequencing technologies), biological batches (that is, organs or species when used over the set of orthologous genes), perturbations such as disease or a combination of these categorical variables. Training a CVAE model with reference studies *S*_1:*N*_ (Fig. 1a) results in a latent space where the effects of condition labels (that is, batch or technology) are regressed out. Thus, we can use this embedding for further downstream analysis such as visualization or identification of cell clusters or subpopulations.

Architectural surgery is a TL approach that takes existing reference models and adapts these to enable query-to-reference mapping. After training an existing autoencoder model on multiple reference datasets, architectural surgery is the process of transferring these trained weights with only minor weight adaptation (fine tuning) and adding a condition node to map a new study into this reference. While this approach is broadly applicable on any deep conditional model, here we apply scArches to three unsupervised models (CVAEs, trVAE, scVI), a semi-supervised (single-cell annotation using variational inference (scANVI))^31^ algorithm and a multimodal (total variational inference (totalVI))^32^ algorithm (Methods).

To facilitate model sharing, we adapted existing reference-building methods to incorporate them into our scArches package as ‘base models’. Reference models built within scArches can be uploaded to a model repository via our built-in application programming interface for Zenodo (Methods). To enable users to map new datasets on top of custom reference atlases, we propose sharing model weights, which one can download from the model repository and fine tune with new query data. This fine tuning extends the model by adding a set of trainable weights per query dataset called ‘adaptors’. In classical conditional neural networks, a study corresponds to an input neuron. As a trained network has a rigid architecture, it does not allow for adding new studies within the given network. To overcome this, we implement the architecture surgery approach to incorporate new study labels as new input nodes (Methods). These new input nodes with trainable weights are the aforementioned adaptors. Importantly, adaptors are shareable, allowing users to further customize shared reference models by downloading a reference atlas, choosing a set of available adaptors for that reference and finally incorporating the user’s own data by training query adaptors (Fig. 1b). Trainable parameters of the query model are restricted to a small subset of weights for query study labels. Depending on the size of this subset, this restriction functions as an inductive bias to prevent the model from strongly adapting its parameters to the query studies. Thus, query data update the reference atlas.

To illustrate the feasibility of this approach, we applied scArches with trVAE, scVI and scANVI (see Supplementary Tables 1–7 for detailed parameters) to consecutively integrate two studies into a pancreas reference atlas comprising three studies (Fig. 1c). To additionally simulate the scenario in which query data contain a new cell type absent in the reference, we removed all alpha cells in the training reference data. We first trained different existing reference models within the scArches framework to integrate training data and construct a reference atlas (Fig. 1d,e and Supplementary Fig. 1, first column). Once the reference atlas was constructed, we fine tuned the reference model with the first query data (SMART-seq2 (SS2)) and iteratively updated the reference atlas with this study (Fig. 1d,e, second column) and the second query data (CelSeq2, Fig. 1d,e, third column). After each update, our model overlays data from all shared cell types present in both query and reference while yielding a separate and well-mixed cluster of alpha cells in the query datasets (black dashed circles in Fig. 1d,e). To further assess the robustness of the approach, we held out two cell types (alpha cells and gamma cells) in the reference data while keeping both in the query datasets. Here our model robustly integrated query data while placing unseen cell types into distinct clusters (Supplementary Fig. 2). Additional testing using simulated data showed that scArches is also robust to simultaneously updating the reference atlas with several query studies at a time (Supplementary Fig. 3).

Overall, TL with architectural surgery enables users to update learnt reference models by integrating query data while accounting for differences in cell type composition.

### Minimal fine tuning performs best for model update

To determine the number of weights to optimize during reference mapping, we evaluated the performance of different fine-tuning strategies. Reference mapping performance was assessed using ten metrics recently established to evaluate data-integration performance^7^ in terms of removal of batch effects and preservation of biological variation. Batch-effect removal was measured via principal-component regression, entropy of batch mixing, *k*-nearest neighbor (kNN) graph connectivity and average silhouette width (ASW). Biological conservation was assessed with global cluster matching (adjusted Rand index (ARI), normalized mutual information (NMI)), local neighborhood conservation (kNN accuracy), cell type ASW and rare cell type metrics (isolated label scores). An accurate reference mapping integration should result in both high conservation of biological variation and high batch-removal scores.

**Fig. 2:**
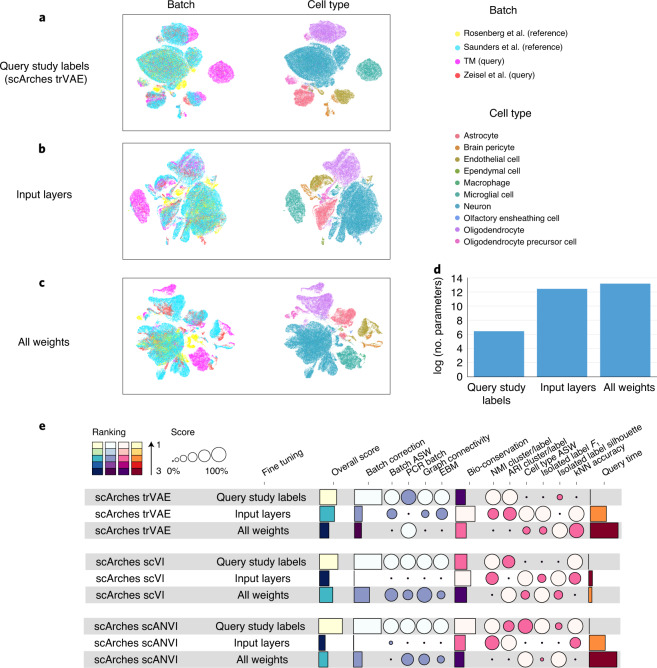
TL and architecture surgery allow fast and accurate reference mapping. **a**–**c**, Comparing different granularity levels in the proposed TL strategy by mapping data from two brain studies to a reference brain atlas. The reference model was trained on a subset of 250,000 cells from two brain studies and then updated with data from Zeisel et al.^35^ and the TM brain subset. Fine-tuning strategies vary from training a few query study label weights (**a**) to input layers of both the encoder and decoder (**b**) and retraining the full network (**c**). **d**, Number of trained weights across these three granularity levels. **e**, Comparison of integration accuracy for different fine-tuning strategies on mapping data from two query studies on a brain reference atlas across various base models. Individual scores are minimum–maximum scaled between 0 and 1. Overall scores were computed using a 40:60-weighted mean of batch correction and bio-conservation scores, respectively (see Methods for further visualization details). EBM, entropy of batch mixing; PCR, principal-component regression.

Next to fine tuning only the weights connecting newly added studies as proposed above (adaptors), we also considered (1) training input layers in both encoder and decoder while the rest of the weights were frozen and (2) fine tuning all weights in the model. We trained a reference model for each base model using 250,000 cells from two mouse brain studies^33,34^. Next, we compared the integration performance of candidate fine-tuning strategies when mapping two query datasets^1,35^ onto the reference data. Applying scArches trVAE to the brain atlas, the model with the fewest parameters performed competitively with other approaches in integrating different batches while preserving distinctions between different cell types (Fig. 2a–c). Notably, the strongly regularized scArches reduced trainable parameters by four to five orders of magnitude (Fig. 2d). Overall, evaluating integration accuracy for different base models demonstrates the optimal time and integration performance trade-off of using adaptors to incorporate new query datasets compared to that of other approaches (Fig. 2e).

### Architectural surgery allows for efficient data integration

**Fig. 3:**
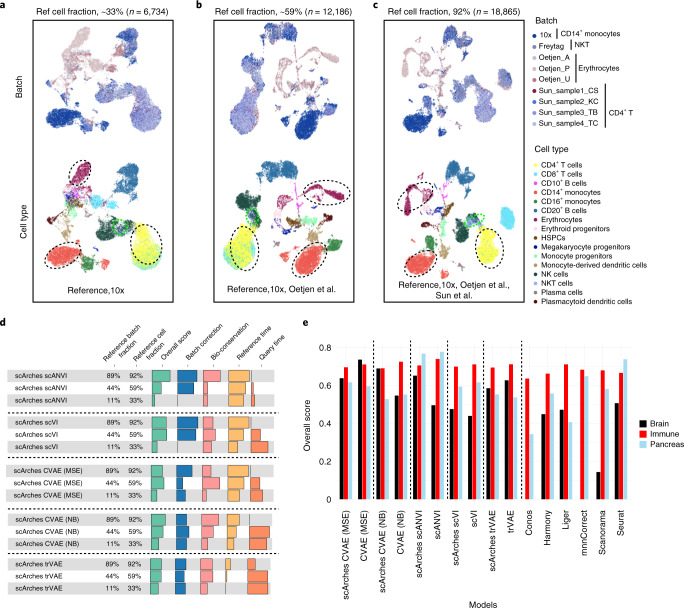
scArches enables efficient reference mapping compared to full integration workflow with existing data-integration methods. **a**–**d**, Evaluating the effect of the reference (ref) size for immune data (*n* = 20,522) on the quality of reference mapping. **a**–**c**, UMAP plots show the latent representation of the integrated query and reference data together for scArches scANVI. Cell types highlighted with dashed circles represent cells unique to a specific study denoted by the batch legend. Reference ratio refers to the fraction of cells in the reference compared to all data. The studies used as reference are indicated at the bottom of each panel. HSPCs, hematopoietic stem and progenitor cells. **d**, Quantitative metrics for the performance of different base models across various reference ratios in immune data. **e**, Comparison of different scArches base models trained with a reference dataset with ~66% of batches in the whole data against de novo full integration methods across immune (*n* = 20,522), pancreas (*n* = 15,681) and brain (*n* = 332,129) datasets. Conos and mnnCorrect were not able to integrate brain data due to excessive memory usage and time requirements, respectively. MSE, mean squared error.

To use scArches, one requires a reference atlas model. The quality of reference mapping performed by scArches relies on the parameterization and architecture chosen for the base model as well as the quality and quantity of reference data. To determine the sensitivity of scArches reference mapping to the reference model used, we investigated how much reference data are needed to enable successful reference mapping. Therefore, we leveraged a human immune cell dataset composed of bone marrow^36^ and peripheral blood mononuclear cells (PBMCs)^37–39^. We built reference models of increasing quality by incrementally including more studies in reference building while using the rest of the studies as query data. To further challenge the model, we included a unique cell type for each study while removing it from the rest of the studies. In our experiments, the reference mapping accuracy of scArches scANVI substantially increased until at least 50% (~10,000 cells) of the data were used as reference (Fig. 3a–c). Specifically, we observed distinct clusters of megakaryocyte progenitors, human pluripotent stem cells, CD10^+^ B cells and erythroid progenitors only in higher reference ratios (Fig. 3b,c), while these were mixed in the lowest reference fraction (Fig. 3a). This observation held true across other base models (Fig. 3d and Supplementary Fig. 4). We repeated similar experiments on brain and pancreas datasets (Supplementary Figs. 5 and 6). Overall, while performance is both model and data dependent, we observed a robust performance when at least 50% of the data, including multiple study batches, are used in reference training (Fig. 3d and Supplementary Figs. 7–10).

Reference mapping is designed to generate an integrated dataset without sharing raw data and with limited computational resources. Thus, it must be evaluated against the gold standard of de novo data integration, for which these restrictions are not present. To assess this, we performed scArches reference mapping using a reference model containing approximately two-thirds of batches and compared this to existing full integration autoencoder methods and other existing approaches^22,40–44^. The overall score for the scArches reference mapping model is similar to that of de novo integration performance (Fig. 3e and Supplementary Figs. 13–15).

We also evaluated the speed of scArches reference mapping compared to full integration strategies. In an scArches pipeline, the reference model must either be built once and can be shared or it can be downloaded directly to map query datasets. Therefore, we consider the time spent by the user to map query datasets as the relevant basis for our comparisons. The running time is also dependent on the base model type. For example, trVAE was much slower than other base models due to the maximum mean discrepancy term, while scVI and scANVI were the fastest (Supplementary Fig. 11a). Overall, scArches can offer a speed-up of up to approximately fivefold and eightfold for scVI and scANVI compared to that of running a de novo autoencoder-based integration for these methods (Supplementary Fig. 16a). This allows mapping of 1 million query cells in less than 1 h (Supplementary Fig. 11b).

### scArches is sensitive to nuanced cell states

We further evaluated scArches under a series of challenging cases. A particular challenge for deep learning methods with many trainable parameters is the small data regime. Thus, we first tested the ability of scArches to map rare cell types. For this purpose, we subsampled a specific cell type in our pancreas and immune integration tasks (delta cells and CD16^+^ monocytes, respectively), such that this population constituted between ~0.1% and ~1.0% of the whole data. Next, we integrated one study as query data and evaluated the quality of reference mapping for the rare cell type. While in all cases the query cells are integrated with reference cells, rare cluster cells can be mixed with other cell types when the fraction is smaller than ~0.5%, and we only observed a distinct cluster for higher fractions (Supplementary Fig. 12).

Second, we evaluated our method on data with continuous trajectories. We trained a reference model using a pancreatic endocrinogenesis dataset^45^ from three early time points (embryonic day (E)12.5, E13.5 and E14.5). We integrated the latest time point (E15.5) as query data. Here query data integrated well with reference data, and our velocity^46^ analysis on the integrated data confirmed the known differentiation trajectory toward major alpha, beta, delta and epsilon fates (Supplementary Fig. 13).

Finally, we evaluated how well scArches resolves nuanced, transcriptionally similar cell types in the query. We therefore trained a reference model excluding natural killer (NK) cells, while the reference data contained highly similar NKT cells. Integrated query and reference cells resulted in a separate NK cluster in proximity to NKT cells (Supplementary Fig. 14a). Repeating a similar experiment with both NK and NKT cells absent in the reference reproduced distinct clusters for both populations in the vicinity of each other (Supplementary Fig. 14b).

### scArches enables knowledge transfer from reference to query

The ultimate goal of query-to-reference mapping is to leverage and transfer information from the reference. This knowledge transfer can be transformative for analyzing new query datasets by transferring discrete cell type labels that facilitate annotation of query data^47,48^ or by imputing continuous information such as unmeasured modalities that are present in reference but absent from query measurements^32,48,49^

We first studied transferring discrete information (for example, cell type labels) to query data. We used the recently published Tabula Senis^3^ as our reference, which includes 155 distinct cell types across 23 tissues and five age groups ranging from 1 month to 30 months from plate-based (SS2) and droplet-based (10x Genomics) assays. As query data, we used cells from the 3-month time point (equivalent to Tabula Muris (TM)).

**Fig. 4:**
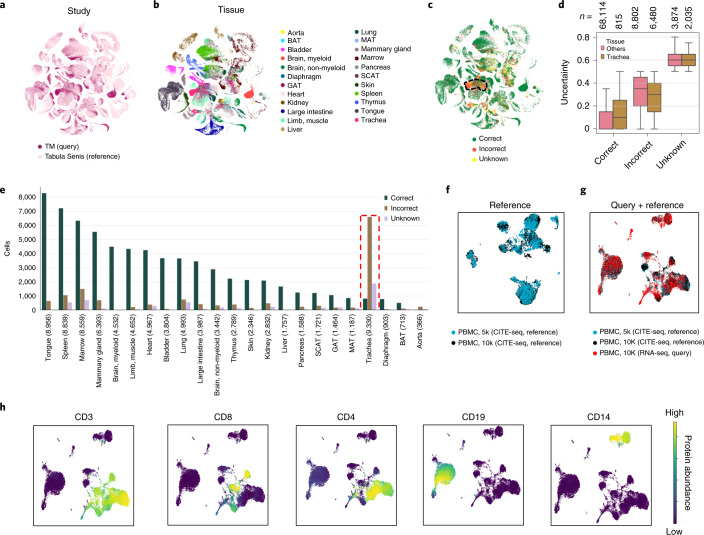
scArches successfully transfers knowledge from reference to query. **a**,**b**, Querying TM (*n* = 90,120) to the larger reference atlas Tabula Senis (*n* = 264,287) using scArches trVAE (**a**) across different tissues (**b**). Tissues were correctly grouped across the two datasets (**a**,**b**). **c**, Location of misclassified and unknown cells after transferring labels from the reference to the query data. The highlighted tissue represents tracheal cells, which we removed from the reference data. **d**, Reported uncertainty of the transferred labels, which was low in correctly classified cells and high in the incorrect and unknown ones, particularly in the trachea. Box plots indicate the median (center lines) and interquartile range (hinges), and whiskers represents minimum and maximum values. Numbers of cells (*n*) are denoted above each box plot. **e**, Numbers of correct, incorrect and unknown cells across different tissues. The red dashed line represents tracheal cells only present in TM. **f**, Construction of a reference CITE-seq atlas using two PBMC datasets (*n* = 10,849 cells). **g**, Integration of scRNA-seq data (*n* = 10,315) into the CITE-seq reference. **h**, Imputation of missing proteins for the query dataset using the reference. BAT, brown adipose tissue; GAT, gonadal adipose tissue; MAT, mesenteric adipose tissue; SCAT, subcutaneous adipose tissue.

The query data consists of 90,120 cells from 24 tissues including a previously unseen tissue, trachea, which we excluded from the reference data. scArches trVAE accurately integrates query and reference data across time points and sequencing technologies and creates a distinct cluster of tracheal cells (*n* = 9,330) (Fig. 4a,b and Supplementary Fig. 15a; see Supplementary Fig. 16 for tissue-level data).

We then investigated the transfer of cell type labels from the reference dataset. Each cell in the query TM was annotated using its closest neighbors in the reference dataset. Additionally, our classification pipeline provides an uncertainty score for each cell while reporting cells with more than 50% uncertainty as unknown (Methods). scArches achieved ~84% accuracy across all tissues (Fig. 4c). Moreover, most of the misclassified cells and cells from the unseen tissue received high uncertainty scores (Fig. 4d and Supplementary Fig. 15b). Overall, classification results across tissues indicated a robust prediction accuracy across most tissues (Fig. 4e), while highlighting cells that were not mappable to the reference. Therefore, scArches can successfully merge large and complex query datasets into reference atlases. Notably, we used scArches to map a large query (the mouse cell atlas^2^) onto TM and further onto a recently published human cell landscape (HCL)^4^ reference, demonstrating applicability to study similarity of cell types across species (Supplementary Note 1 and Supplementary Figs. 18–21). Overall, scArches-based label projection performs competitively when compared with state-of-the-art methods such as SVM rejection^47,50^, Seurat version 3 (ref. ^22^) and logistic regression classifiers^50^ (Supplementary Fig. 17).

In addition to the label transfer, one can use reference atlases to impute continuous information in the query data such as missing antibody panels in RNA-seq-only assays. Indeed, one can combine scArches with existing multimodal integration architectures such as totalVI^32^, a model for joint modeling of RNA expression and surface protein abundance in single cells. Leveraging scArches totalVI, we built a cellular indexing of transcriptomes and epitopes by sequencing (CITE-seq)^51^ reference using two publicly available PBMC datasets (Fig. 4f). Next, we integrated query scRNA-seq data into the reference atlas (Fig. 4g) and used the multimodal reference atlas to impute missing protein data for the query dataset. Using imputed protein abundances, we can distinguish the observed major populations such as T cells (CD3^+^, CD4^+^ and CD8^+^), B cells (CD19^+^) and monocytes (CD14^+^) (Fig. 4h) (see Supplementary Fig. 22 for all proteins).

### Preserving COVID-19 cell states after reference mapping

**Fig. 5:**
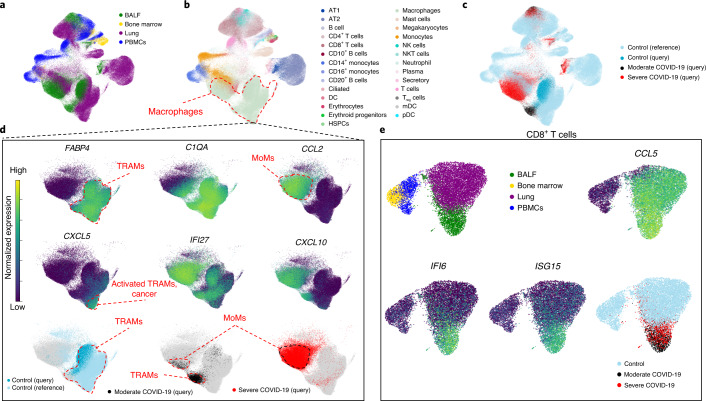
scArches resolves severity in COVID-19 query data mapped to a healthy reference and reveals emergent cell states. **a**–**c**, Integration of query data from immune and epithelial cells from patients with COVID-19 on top of a healthy immune atlas across multiple tissues (**a**), cell types (**b**) and cell states (**c**). BALF, bronchoalveolar lavage fluid; DC, dendritic cell; T_reg_ cells, regulatory T cells. **d**, Comparison of various macrophage subpopulations across both healthy and COVID-19 states. Top, TRAMs are characterized by expression of *FABP4*, while monocyte-derived inflammatory macrophages (MoMs) are characterized by expression of *CCL2*. Upregulation of *C1QA* illustrates maturation of MoMs as they differentiate from monocytes to macrophages. Middle, *CXCL5*, *IFI27* and *CXCL10* illustrate context-dependent activation of TRAMs. Bottom, scArches correctly maps TRAMs from query to TRAMs from reference, while preserving MoMs, unseen in the reference, as a distinct cell type. **e**, Separation of activated query CD8^+^ T cells from patients with COVID-19 from the rest of CD8^+^ T cells in the reference. AT, alveolar type; mDC, myeloid dendritic cells; pDC, plasmacytoid dendritic cells.

In the study of disease, contextualization with healthy reference data is essential. A successful disease-to-healthy data integration should satisfy three criteria: (1) preservation of biological variation of healthy cell states; (2) integration of matching cell types between healthy reference and disease query; and (3) preservation of distinct disease variation, such as the emergence of new cell types that are unseen during healthy reference building. To showcase how one can perform disease contextualization with scArches, we created a reference aggregated from bone marrow^36^, PBMCs^37–39^ and normal lung tissue^52–54^ (*n* = 154,723; Fig. 5a–c) and then mapped onto it a dataset containing alveolar macrophages and other immune cells collected via bronchoalveolar lavage from (1) healthy controls and patients with (2) moderate and (3) severe COVID-19 (*n* = 62,469)^55^. As described by Liao and colleagues, this dataset contains immune cells found in the normal lung (for example, tissue-resident alveolar macrophages, TRAMs) as well as unique populations that are absent in the normal lung and emerge only during inflammation (for example, monocyte-derived alveolar macrophages, MoAMs)^55^. We used a negative binomial (NB) CVAE base model for this experiment (Methods).

We first evaluated the integration of query batches in the reference. scArches successfully integrated alveolar macrophages from different datasets and preserved biological variability between them, although some ambient RNA signals remained (Supplementary Note 2 and Supplementary Fig. 23). For example, activated TRAMs (*FABP4*^+^*IL1B*^+^*CXCL5*^+^) that originate from a single individual (donor 2 in the Travaglini et al.^52^ dataset) formed a distinct subcluster within TRAMs (Fig. 5a–d). We then evaluated the projection of COVID-19 query data onto the reference model. The dataset from Liao and colleagues contains the following cell types: airway epithelial cells, plasma cells and B cells, CD4^+^ and CD8^+^ T cells, NK cells, neutrophils, mast cells, dendritic cells, monocytes and alveolar macrophages (Fig. 5b,c and Supplementary Fig. 24)^55^. Within the macrophage cluster (characterized by the expression of *C1QA*), two distinct populations dominated the structure of the embedding (Fig. 5c,d): TRAMs (*FABP4*^+^*C1Q*^+^*CCL2*^−^) and inflammatory MoAMs (*FABP4*^−^*C1Q*^+^*CCL2*^+^). As expected, query TRAMs from healthy controls integrated well with TRAMs from the reference dataset. While TRAMs from patients with moderate COVID-19 integrated with TRAMs from control lung tissue, they did not mix with normal TRAMs completely, as they were activated and characterized by increased expression of *IFI27* and *CXCL10*. MoAMs are predominantly found in samples from patients with severe COVID-19 and to a lesser extent in samples from patients with moderate COVID-19. MoAMs originate from monocytes that are recruited to sites of infection (as illustrated by the gradient of *C1QA* expression) and thus do not appear in healthy reference tissue. Indeed, MoAMs were embedded in closer proximity to monocytes than to TRAMs in our embedding, reflecting their ontological relationship (see Supplementary Fig. 25 for partition-based graph abstraction^56^ proximity analysis).

We then evaluated CD8^+^ T cells. While the reference bone marrow and blood cells predominantly contained naive CD8^+^ T cells (*CCL5*^−^), lung and bronchoalveolar lavage fluid contained cytotoxic memory CD8^+^ T cells (*GZMA*^+^*GZMH*^+^; Fig. 5e and Supplementary Fig. 26). Moreover, cytotoxic memory CD8^+^ T cells from patients with COVID-19 were characterized by the expression of interferon-response genes *ISG15*, *MX1* and others, which is in agreement with a recent report that the interferon response is a feature separating severe acute respiratory syndrome coronavirus 2 pneumonia from other viral and non-viral pneumonias^57,58^ (Fig. 5e, Supplementary Note 2 and Supplementary Fig. 26).

Overall, the scArches joint embedding was dominated by nuanced biological variation, for example, macrophage subtypes, even when these subtypes were not annotated in reference datasets (for example, activated TRAMs from patients with moderate COVID-19 or a patient with a lung tumor). Although disease states were absent in the reference data, scArches separated these states from the healthy reference and even preserved biological variation patterns. Hence, disease-to-healthy integration with scArches met all three criteria for successful integration.

## Discussion

We introduced architectural surgery, an easy-to-implement approach for TL, reusing neural network models by adding input nodes and weights (adaptors) for new studies and then fine tuning only those parameters. Architectural surgery can extend any conditional neural network-based data-integration method to enable decentralized reference updating, facilitate model reuse and provide a framework for learning from reference data.

In applications, we demonstrated how integration of whole-species atlases enables the transfer of cell type annotations from a reference to a query atlas. We further showed that COVID-19 query data can be mapped on top of a healthy reference while retaining variation among both disease and healthy states, which we promote in scArches by avoiding showing the method the disease effect during training. In general, different effects such as disease states are assumed to be orthogonal in high-dimensional space^43^; thus, if a batch-confounded effect (for example, any donor-level covariate when donor is used as batch) is not seen during training, we would not expect it to be removed. We observe this phenomenon in our COVID-19 example and in multiple experiments: biologically meaningful variations from held-out alpha cells in the pancreas (Fig. 1d,e) or unseen nuanced cell identities in immune cell data (Supplementary Fig. 14) are mapped to a new location when they are unseen during training.

The reduction in model training complexity by training adaptors moreover leads to an increase in speed while preserving integration accuracy when compared to full integration methods. It also improves usability and interpretability, because mapping a query dataset to a reference requires no further hyperparameter optimization and keeps reference representation intact. Adaptors only impact the first network layer and therefore ‘commute’: application order is irrelevant for iteratively expanding a reference, arriving always at the same result due to the frozen nature of the network and independence of adaptor weights. With scArches, one can therefore use pre-trained neural network models without computational expertise or graphics processing unit power to map, for example, disease data onto stored reference networks prepared from independent atlases. We make use of these features by providing a model database on Zenodo (Methods).

Model sharing in combination with reference mapping via scArches allows users to create custom reference atlases by updating public ones and paves the way for automated and standardized analyses of single-cell studies. Especially for human data, sharing expression profiles is often difficult due to data-protection regulations, size, complexity and other organizational hurdles. With scArches, users can obtain an overview of the whole dataset to validate harmonized cell type annotation. By sharing a pre-trained neural network model that can be locally updated, international consortia can generate a joint embedding without requiring access to the full gene sets. In turn, users can quickly build upon this by mapping their own typically much smaller data into the reference, acquiring robust latent spaces, cell type annotation and identification of subtle state-specific differences with respect to the reference.

scArches is a tool that leverages existing conditional autoencoder models to perform reference mapping. Thus, by design, it inherits both benefits and limitations of the underlying base models. For example, a limitation of these models is that the integrated output is a low-dimensional latent space instead of a corrected feature matrix as provided by mnnCorrect or Scanorama. While generating a batch-corrected input matrix is possible^30^, this may lead to spurious and false signals similar to denoising methods^59^. Similarly, imputation of modalities not measured in query data (for example, via scArches totalVI) performs better for more abundant features, which has already been outlined in the original totalVI publication^32^. A further limitation is the need for a sufficiently large and diverse set of samples for reference building. Deep learning models typically have more trainable parameters than other integration methods and thus often require more data. This constraint translates directly to the performance of scArches reference mapping (Fig. 3a–d): using a small reference along with a low number of studies leads to poor integration of query data while removing biological variation such as nuanced cell types. Furthermore, even with equal training data, reference model performance will differ, affecting reference mapping via scArches. As robust and scalable reference building is still ongoing research in the scRNA-seq field^7^, the choice of reference model is a central challenge when using scArches. Yet we demonstrate that even imperfect reference models (Supplementary Note 3) can be used for meaningful analyses as demonstrated by our data analysis of patients with COVID-19. Finally, one must consider the limitations of the base model on batch-effect removal during reference mapping, in which it is unlikely to remove stronger batch effects than those seen in the training data. In our cross-species experiments, reference mapping performs well mostly in immune cell populations, which appear to contain the smallest batch effect across species (Supplementary Figs. 18, 20 and 21).

While scArches is applicable in many scenarios, it is best suited when the query data consists of cell types and experimental protocols similar to the reference data. Then, the query data may easily contain new cell types or states such as disease or other kinds of perturbations, which are preserved after mapping. Additionally, we advise against using scArches for integrating query data with a reference created out of a single study and recommend integration with full sample access instead. Further, the number of overlapping genes between query and reference data can also influence integration quality. We generally recommend using a larger set of highly variable genes (HVGs) in the reference-building step to guarantee a bigger feature overlap between reference and query, which increases the robustness of reference mapping in the presence of missing genes (Methods and Supplementary Fig. 28).

We envision two major directions for further applications and development. First, scArches can be applied to generate context-specific large-scale disease atlases. Large disease reference datasets are increasingly becoming available^60–62^. By mapping between disease references, we can assess the similarity of these diseases at the single-cell level and thus inform for finding mechanisms, reverting disease state or studying perturbations, for example, for drug repurposing. The suitability of model organisms for disease research can be directly translated into the human context: for example, projecting mouse single-cell tumor data on a reference human patient tumor atlas may help to identify accurate tumor models that include desired molecular and cellular properties of a patient’s microenvironment. Incorporating additional covariates as conditional neurons in the reference model will allow modeling of treatment response with a certain perturbation or drug^63,64^. Secondly, we envision assembling multimodal single-cell reference atlases to include epigenomic^65^, chromosome conformation^66^, proteome^51^ and spatially resolved measurements.

In summary, with the availability of reference atlases, we expect scArches to accelerate the use of these atlases to analyze query datasets.

## Methods

### Architecture surgery

Our method relies on a concept known as TL. TL is an approach in which weights from a model trained on one task are taken and used as weight initialization or fine tuning for another task. We introduce an architecture surgery, a strategy to apply TL in the context of conditional generative models and single-cell data. Our proposed method is general and can be used to perform TL on both CVAEs and conditional generative adversarial nets^67^.

Let us assume that we want to train a reference CVAE model with a d-dimensional dataset (*x* ϵ *R*^*d*^) from *n* different studies (*s* ϵ *R*^*n*^), where *R* denotes real number space. We further assume that the bottleneck *z* with layer size is *k*(*z* *ϵ* *R*^*k*^). Then, an input for a single cell *i* will be *x*′ = *x* · *s*, where *x* and *s* are the *d*-dimensional gene expression profile and *n*-dimensional one-hot encoding of study labels, respectively. The · symbol denotes the row-wise concatenation operation. Therefore, the model receives (*d* + *n*)-dimensional and (*k* + *n*)-dimensional vectors as inputs for encoder and decoder, respectively. Assuming *m* query datasets, the target model will be initialized with all the parameters from the reference model. To incorporate *m* new study labels, we add *m* new dimensions to *s* in both encoder and decoder networks. We refer to these new added study labels as *s*′. Next, *m* new randomly initialized weight vectors are also added to the first layer of the encoder and decoder. Finally, we fine tune the new model by only training the weights connected to the last *m* dimensions of *x*′ that correspond to the condition labels. Let us assume that *p* and *q* are the number of neurons in the first layer of the encoder and decoder; then, during the fine tuning, only (*m*) × (*p* + *q*) parameters will be trained. Let us parameterize the first layer of the encoder and decoder part of the scArches as *f*_1_ and *g*_1_, respectively. Let us further assume that ReLU activations are used in the layers. Therefore the equations for *f*_1_ and *g*_1_ are

$$\begin{array}{l}f_1(x,s,s\prime ;\phi _x,\phi _s,\phi _{s\prime }) = {\textrm{max}}(0,\phi _x^Tx + \phi _s^Ts + \phi _{s\prime }^Ts\prime )\\ g_1(z,s,s\prime ;\theta _z,\theta _s,\theta _{s\prime }) = {\textrm{max}}(0,\theta _z^Tz + \theta _s^Ts + \theta _{s\prime }^Ts\prime ),\end{array}$$

where *ϕ* and *θ* are parameters of encoder and decoder, and *T* denotes transpose operation. Therefore, the gradients of *f* and *g* with respect to *ϕ*_*s*′_ and *θ*_*s*′_ are

$$\begin{array}{l}\nabla_{\phi_{s'}}f_1 = \left\{\begin{array}{lr} 0 & {\mathrm{if}}\ {\phi_x^Tx + \phi_s^Ts + \phi_{s'}^Ts'} {\le} 0\\ s' & {\mathrm{otherwise}}\ \end{array} \right. \\ \nabla_{\theta_{s'}}g_1 = \left\{\begin{array}{lr} 0 & {\mathrm{if}}\ {\theta_z^Tz + \theta_s^Ts + \theta_{s'}^Ts'} {\le} 0\\ s' & {\mathrm{otherwise}} \end{array} \right. \end{array}$$

Finally, because all other weights except *ϕ*_*s*′_ and *θ*_*s*′_ are frozen, we only compute the gradient of scArches’ cost function with respect to *ϕ*_*s*′_ and *θ*_*s*′_:

$$\begin{array}{l}\nabla _{\phi _{s\prime }}L_{\textrm{scArches}}(x,s,s\prime ;\theta ,\phi ) = \nabla _{f_1}L_{\textrm{scArches}}(x,s,s\prime ;\phi ) \cdot \nabla _{\phi _{s\prime }}f_1(x,s,s\prime ;\phi _x,\phi _s,\phi _{s\prime })\\ \nabla _{\theta _{s\prime }}L_{\textrm{scArches}}(x,s,s\prime ;\theta ,\phi ) = \nabla _{g_1}L_{\textrm{scArches}}(z,s,s\prime ;\theta ,\phi ) \cdot \nabla _{\theta _{s\prime }}g_1(x,s,s\prime ;\theta _z,\theta _s,\theta _{s\prime }).\end{array}$$

### scArches base models

#### Conditional variational autoencoders

Variational autoencoders (VAEs)^68^ were shown to learn the underlying complex structure of data. VAEs were proposed for generative modeling of the underlying data leveraging variational inference and neural networks to maximize the following equation:

$$p_\theta (X\mid S) = {\int} {p_\theta } (X\mid Z,S)p_\theta (Z\mid S)dZ,$$

where *X* is a random variable representing the model’s input, *S* is a random variable indicating various conditions, *θ* is the neural network parameters, and $p_\theta (X\mid Z,S)$ is the output distribution that we sample *Z* to reconstruct *X*. In the following equation, we exploit notations from ref. ^29^ and a tutorial from ref. ^69^. We approximate the posterior distribution $p_\theta (Z|X,S)$ using the variational distribution $q_\phi (Z|X,S)$ that is approximated by a deep neural network parameterized with *ϕ*:

$$\begin{aligned}L_{\textrm{CVAE}}(X,S;\phi ,\theta ) &= \log p_\theta (X\mid S) - \alpha \cdot D_{\textrm{KL}}(q_\phi (Z|X,S)||p_\theta (Z|X,S)) = \\ &= \mathbb{E}_{q_\phi (Z\mid X,S)}[\log p_\theta (X\mid Z,S)] - \alpha \cdot D_{\textrm{KL}}(q_\phi (Z|X,S)||p_\theta (Z|S)),\end{aligned}$$

where $\theta = \{ \theta \prime ,\theta _z,\theta _s\}$ and $\phi = \{ \phi \prime ,\phi _x,\phi _s\}$ are parameters of decoder and encoder, respectively, ${\mathbb{E}}$ is the expectation and *D*_KL_ is the Kullback-Leibler divergence scaled by parameter α. On the left-hand side, we have the log likelihood of the data and an error term that depends on the capacity of the model. The right-hand side of the above equation is also known as the evidence lower bound. CVAE^70^ is an extension of VAE framework in which $S \ne \emptyset$.

#### scArches trVAE

trVAE^30^ builds upon VAE^68^ with an extra regularization to further match the distribution between conditions. Following the method proposed by Lotfollahi et al.^30^, we use the representation of the first layer in the decoder, which is regularized by maximum mean discrepancy^71^. For implementation, we use multi-scale radial basis function (RBF) kernels defined as

$$k\left( { x,x\prime } \right) = \mathop {\sum }\limits_{i = 1}^l k\left( {x,x\prime ,\gamma _i} \right),$$

where $k\left( {x,x\prime ,\gamma _i} \right) = e^{ - \gamma _i| \ast x - \ast x\prime |^2}$, *γ*_*i*_ is a hyperparameter, and *l* denotes maximum number of RBF kernels.

We will parameterize the encoder and decoder part of scArches as *f*_*ϕ*_ and *g*_*θ*_, respectively. So the networks *f*_*ϕ*_ and *g*_*θ*_ will accept inputs *x*, *s* and *z*, *s*, respectively. Let us distinguish the first ($g_{\theta _z,\theta _s}^{(1)}$) and the remaining layers ($g_{\theta \prime }^{(2)}$) of the decoder network $g_\theta = g_{\theta \prime }^{(2)} \circ g_{\theta _z,\theta _s}^{(1)}$. Therefore, we can define the following maximum mean discrepancy (MMD) cost function:

$$L_{\textrm{MMD}}(X,S;\phi ,\theta _z,\theta _s) = \mathop {\sum }\limits_{i \ne j}^{\ {\textrm{No. studies}}} l_{\textrm{MMD}}(g_{\theta _z,\theta _s}^{(1)}(f_\phi (X_{S = i},i),i),g_{\theta _z,\theta _s}^{(1)}(f_\phi (X_{S = j},j),j)),$$

where

$$\begin{array}{rcl}l_{\textrm{MMD}}(X,X^\prime ) & = & \frac{1}{{N_0^2}}\mathop {\sum }\limits_{n = 1}^{N_0} \mathop {\sum }\limits_{m = 1}^{N_0} k(x_n,x_m) \\ && + \frac{1}{{N_1^2}}\mathop {\sum }\limits_{n = 1}^{N_1} \mathop {\sum }\limits_{m = 1}^{N_1} k(x_n^\prime ,x_m^\prime ) - \frac{2}{{N_0N_1}}\mathop {\sum }\limits_{n = 1}^{N_0} \mathop {\sum }\limits_{m = 0}^{N_1} k(x_n,x_m^\prime ).\end{array}$$

We used the notation *X*_*S*=*i*_ for samples drawn from *i*th study distribution in the training data. Finally, the trVAE’s cost function is

$$L_{\textrm{trVAE}}(X,S;\phi ,\theta ) = L_{\textrm{CVAE}}(X,S;\phi ,\theta ) - \beta \cdot L_{\textrm{MMD}}(X,S;\phi ,\theta _z,\theta _s),$$

where *β* is a regularization scale parameter. The gradients of trVAE’s cost function with respect to *ϕ*_*s*_ and *θ*_*s*_ are

$$\begin{array}{l}\nabla _{\phi _s}L_{\textrm{trVAE}}(X,S;\theta ,\phi ) = \nabla _{\phi _s}L_{\textrm{CVAE}}(X,S;\theta ,\phi ) - \beta \cdot \nabla _{\phi _s}L_{\textrm{MMD}}(X,S;\phi ,\theta _z,\theta _s),\\ \nabla _{\theta _s}L_{\textrm{trVAE}}(X,S;\theta ,\phi ) = \nabla _{\theta _s}L_{\textrm{CVAE}}(X,S;\theta ,\phi ) - \beta \cdot \nabla _{\theta _s}L_{\textrm{MMD}}(X,S;\phi ,\theta _z,\theta _s).\end{array}$$

Therefore *L*_trVAE_ can be optimized using stochastic gradient ascent with respect to *ϕ*_*s*_ and *θ*_*s*_ as all the other parameters are frozen.

#### scArches scVI

Lopez et al.^27^ developed a fully probabilistic approach, called scVI, for normalization and analysis of scRNA-seq data. scVI is also based on a CVAE, described in detail above. But, in contrast to the trVAE architecture, the decoder assumes a zero-inflated negative binomial (ZINB) distribution; and therefore the reconstruction loss differs to the MSE loss of trVAE. Another major difference is that scVI explicitly models the library size, which is needed for the ZINB loss calculation with another shallow neural network called the library encoder. Therefore, with similar notation as above, we have the output distribution $p(X|Z,S,L)$, where *L* is the scaling factor that is sampled by the outputs of the library encoder, namely the empirical mean *L*_*μ*_ and the variance *L*_*σ*_ of the log library per batch:

$$L \sim {\textrm{lognormal}}(L_\mu ,L_\sigma ^2).$$

When we now separate the outputs of the decoder *g*_*θ*_ into $g_\theta ^x$, the decoded mean proportion of the expression data, and $g_\theta ^d$, the decoded dropout effects, we can write the ZINB mass function for $p(X|Z,S,L)$ in the following closed form:

$$\left\{\begin{array}{l}\\ p(X = 0 | Z, S, L) = \\ \qquad g_{\theta}^d(Z, S) + (1 - g_{\theta}^d(Z, S))\left(\frac{\Sigma}{\Sigma + L \cdot g_{\theta}^x(Z,S)} \right)^{\Sigma} \\p(X = Y | Z, S, L) =\\ \qquad (1 - g_{\theta}^d(Z,S))\frac{\Gamma(Y + \Sigma)}{\Gamma(Y+1)\Gamma(\Sigma)}\left(\frac{\Sigma}{\Sigma + L \cdot g_{\theta}^x(Z,S)}\right)^{\Sigma}\left(\frac{g_{\theta}^x(Z,S)}{\Sigma + L \cdot g_{\theta}^x(Z, S)}\right)^{Y},\end{array}\right.$$

where Σ is the gene-specific inverse dispersion, Γ is the gamma function, and *Y* represents non-zero entries drawn from a ZINB distribution. Because the evidence lower bound and therefore the optimization objective can be calculated by applying the reparameterization trick and supposing Gaussians, which is possible here because of the proposed ZINB distribution, we can write the scVI cost function as follows:

$$L_{\textrm{scVI}}(X,S;\phi ,\theta ) = L_{\textrm{CVAE}}(X,S;\phi ,\theta ) - \alpha \cdot D_{\textrm{KL}}(q_\phi (L|X,S)||p_\theta (L)).$$

Furthermore, because of the applied reparameterization trick, an automatic differentiation operator can be used, and the cost function can be optimized by applying stochastic gradient descent. For the application in scArches, we removed the library encoder and computed the library size for each batch in a closed form by summing up the counts. This does not decrease the performance of the model and accelerates the surgery step. The resulting network can then be used similarly to the trVAE network by simply retraining only the condition weights corresponding to the new batch annotations in *S*.

#### scArches scANVI

scANVI is a semi-supervised method that builds up on the scVI model and was proposed in detail by Xu et al.^31^. By constructing a mixture model, it is able to make use of any cell type annotations during autoencoder training to improve latent representation of the data. In addition to this, scANVI is capable of labeling datasets with only some marker gene labels as well as transferring labels from a labeled dataset to an unlabeled dataset. For the training of scANVI, the authors proposed an alternating optimization of the cost function $L_{\textrm{scANVI}}(X,S;\phi ,\theta )$ and the classification loss *C*, which results from a shallow neural network that serves as a classifier with a cross-entropy loss after the last softmax layer. In more detail, the cost function can be formulated in the following manner:

$$L_{\textrm{scANVI}}(X,S;\phi ,\theta ) = L_{\textrm{labeled}}(X,S,C;\phi ,\theta ) + L_{\textrm{unlabeled}}(X,S;\phi ,\theta ),$$

where *C* is the cell types in the annotated datasets, and both cost function summands *L*_labeled_ and *L*_unlabeled_ are obtained by similar calculations as in the case of scVI. The major difference here, however, is that the Kullback–Leibler divergence is applied to an additional latent encoder that takes cell type annotations into account. For the unlabeled case, each sample is broadcasted into every available cell type. As scANVI builds up on scVI, we use the same adjustments here to apply surgery. On top of that, we also freeze the classifier even for semi-supervised query data, because we want an unchanging reference performance for building a cell atlas and also to force cells in the query data with the same cell type annotation to be near to the corresponding reference cells in the latent representation.

#### scArches totalVI

For the purpose of combining paired measurement of RNA and surface proteins from the same cells, such as for CITE-seq data, Gayoso et al.^32^ presented a deep generative model called totalVI. totalVI learns a joint low-dimensional probabilistic representation of RNA and protein measurements. For the RNA portion of the data, totalVI uses an architecture similar to that of scVI, which we discussed in detail above; but, for proteins, a new model is introduced that separates protein information into background and foreground components. With the surgery functionality of scArches added to totalVI, it is now possible to learn a joint latent space of RNA and protein data on a CITE-seq reference dataset and do surgery on a query dataset with only RNA data to impute protein data for that query dataset as well. To accomplish this goal, we again only retrain the weights that correspond to the new batch labels.

#### CVAEs for single-cell genomics

CVAEs were first applied to scRNA-seq data in scVI^29^ for data integration and differential testing. Here we focus on how CVAEs perform data integration and potential pitfalls. These models receive a matrix of gene expression profile for cells (*X*) and label (condition) matrix (*S*). The condition matrix comprises a nuisance variable, which we want to regress out from the data. Labels can encode batch, technologies, disease state or other discrete variables. The CVAE model seeks to infer a low-dimensional latent space (*Z*) for the cell that would be free of variations explained by the label variable. For example, if the labels are the experimental batches, then similar cell type separated by batch effect in the original gene expression space will be aligned together. Importantly, variation attributed to the labels will be merely regressed in the latent space while still present in the output of the CVAE. Therefore, the reconstructed output will still contain batch effects. Additionally, while autoencoder-based data-integration methods were shown to perform best when outputting integrated embeddings, these methods can also output corrected expression matrices. This is achieved by forcing all batches to be transformed to a specific batch as previously shown in scGen.

scArches builds upon existing CVAEs. The results of the integration heavily depend on the type of labels used as batch covariates for condition inputs. If the dataset is the batch covariate, within-dataset donor effects will not be removed, but donors become more comparable across datasets. In our COVID-19 example, the disease is used as a query and thus is not captured fully in the encoder, which is trained on data from healthy individuals. Adaptor training removes the donor- and/or dataset-specific batch effect from a disease sample but does not remove variation unseen in network training. Thus, choice of training data and choice of batch covariate are crucial to assess whether variation from disease is removed in training or not.

Overall, the choice and design of the label matrix is a crucial step for optimal outcome. The label matrix can encode one covariate (for example, batch), multiple covariates (for example, technology, cell types, disease, species,…) or a combination of covariates (for example, technology and species). However, the interpretability of the latent space will be challenging in the presence of complex label design and will require extra caution.

#### Model sharing

We currently support an application programming interface to upload and download model weights and data (if available) using Zenodo. Zenodo is a general-purpose open-access repository developed to enable researchers to share datasets and software. We have provided step-by-step guides for the whole pipeline from training and uploading models to downloading, updating and further sharing models. These tutorials can be found in the scArches GitHub repository (https://github.com/theislab/scarches).

#### Feature overlap between reference and query

An important practical challenge for reference mapping using scArches is the number of features (genes) that are shared between the query and the reference model and/or dataset. It is important to note that, with the current pipeline, the query data must have the same gene set as the reference model. Therefore, the user has to replace missing reference genes in the query with zeros. We investigated the effect of zero filling and observed that integration performance was robust when 10% (of 2,000 genes) were missing from query data. However, the performance will deteriorate with larger differences between query and reference (Supplementary Fig. 28a). We further observed good integration with 4,000 HVGs, even when 25% of genes were missing from the query data, conveying that the model would be robust if the overall number of shared genes is large (for example, 4,000 HVGs, Supplementary Fig. 28b).

### Evaluation metrics

Evaluation metrics and their definitions in the current paper were taken from work by Luecken et al.^7^, unless specifically stated otherwise.

#### Entropy of batch mixing

This metric^43^ works by constructing a fix similarity matrix for cells. The entropy of mixing in a region of cells with *c* batches is defined as

$$E = \mathop {\sum }\limits_{i = 1}^c p_i\log_c(p_i),$$

where _*pi*_ is defined below as

$$p_i = \frac{{\ {\textrm{no. cells with batch}}\,i\,{\textrm{in the region}}}}{{\ {\textrm{no. cells in the region}}}}.$$

Next, we define *U*, a uniform random variable on the cell population. Let *B*_*U*_ be the frequencies of 15 nearest neighbors for the cell *U* in batch *x*. We report the entropy of this variable and then average across *T* = 100 measurements of *U*. To normalize the entropy of the batch mixing score between 0 and 1, we set the base of the logarithm to the number of batches *c*.

#### Average silhouette width

Silhouette width measures the relationship between within-cluster distances of a cell and between-cluster distances of that cell to the closest cluster. In general, an ASW score of 1 implies clusters that are well separated, an ASW score of 0 implies overlapping clusters, and an ASW score of −1 implies strong misclassification. When we use the ASW score as a measure of biological variance, we calculate it on cell types in the following manner:

$${\textrm{ASW}}_c = \frac{{\textrm{ASW} + 1}}{2},$$

where the final score is already scaled between 0 and 1. Therefore larger values correspond to denser clusters. In contrast to the ASW_*c*_ score, we also calculate an ASW score on batches within cell clusters to obtain a measure for batch-effect removal. In this case, we again scale but also invert the ASW score to have a consistent metric comparison:

$${\textrm{ASW}}_b = 1 - {\textrm{abs}}({\textrm{ASW}}).$$

A higher final score here implies better mixing and therefore a better batch-removal effect.

#### Normalized mutual information

We use NMI to compare the overlap of two different cell type clusterings. In detail, we computed a Louvain clustering on the latent representation of the data and compared it to the latent representation itself in a cell type-wise manner. To obtain scores between 0 and 1, the overlap was scaled using the mean of entropy terms for cell type and cluster labels. Therefore an NMI score of 1 corresponds to a perfect match and good conservation of biological variance, whereas an NMI score of 0 corresponds to uncorrelated clustering.

#### Adjusted Rand index

This metric considers correct clustering overlaps as well as counting correct disagreements between two clusterings. Again, similar to NMI, cell type labels in the integrated dataset are compared with Louvain clustering. The adjusted Rand index score is normalized between 0 and 1, where 1 corresponds to good conservation of biological variance and 0 corresponds to random labeling.

#### Principal-component regression

In contrast to principal-component analysis (PCA), we calculate a linear regression *R* with respect to the batch label onto each principal component. The total variance (Var) explained by the batch variable can then be formulated as follows:

$${\textrm{Var}}(X|B) = \mathop {\sum }\limits_{i = 1}^N {\textrm{Var}}(X|{\textrm{PC}}_i) \cdot R^2({\textrm{PC}}_i|B),$$

where *X* is the data matrix, *B* is the batch label, and *N* is the number of principal components (PC).

#### Graph connectivity

For this metric, we calculate a subset kNN graph $G(N_c,E_c)$ for each cell type label *c*, such that each subset only contains cells from the given label. The total graph connectivity score can then be calculated as follows:

$$gc = \frac{1}{{|C|}}\mathop {\sum}\limits_{c \in C} {\frac{{|{\textrm{LCC}}(G(N_c,E_c))|}}{{|N_c|}}} ,$$

where *C* is the set of cell type labels, $|{\textrm{LCC}}()|$ is the number of nodes in the largest connected component of the graph, and |*N*_*c*_| is the number of nodes with the given cell type label. This means that we check if the graph representation of the latent representation connects all cells with the same cell type label. Therefore, a score of 1 would imply that all cells with the same cell type label are connected, which would further indicate good batch mixing. A graph in which no cells are connected would result in a score of 0.

#### Isolated label *F*_1_

We defined isolated labels as cell type labels that are present in the least number of batches. If there are multiple isolated labels, we simply take the mean of each score. To determine how well those cell types are separated from other cell types in the latent representation, we first determine the cluster with the largest number of an isolated label. Subsequently, an *F*_1_ score of the isolated label against all other labels within that cluster is computed, where the *F*_1_ score is defined as follows:

$$F_1 = 2\frac{{{\textrm{precision}} \cdot {\textrm{recall}}}}{{\textrm{precision} + {\textrm{recall}}}}.$$

This results in a score between 0 and 1 once again, where 1 implies that all cells with the isolated label are captured in the cluster.

#### Isolated label silhouette

For this metric, we use ASW_*c*_, defined above, but only on the isolated label subset of the latent representation. Scaling and meaning of the score are the same as described for ASW. If there are multiple isolated labels, we average over each score similar to the isolated labeled *F*_1_ score.

#### kNN accuracy

We first compute the 15 nearest neighbors of each cell in the data. We then compute the ratio of the correct cell type annotations inside those 15 neighbors. This cell-wise score is then averaged over all cell types separately and then averaged over all remaining scores again to obtain a single kNN-accuracy score between 0 and 1. A higher kNN-accuracy score corresponds to better preservation of local cell type purity. This metric was inspired by a similar metric used in scANVI.

### Visualization of integration scores

To compare performances of different models, we designed an overview table (inspired by Saelens et al.^72^) that displays individual integration scores as circles and aggregated scores as bars. Each individual score is minimum–maximum scaled to improve visual comparison of different models and then averaged into aggregated scores by category (batch correction and biological conservation). Finally, an overall score is calculated as a weighted sum of batch correction and bio-conservation, considering a ratio of 40:60, respectively. When shown, reference and query times are not considered in the calculation of aggregated scores. Moreover, these time values are scaled together to allow direct comparison. The overall ranking of each model, for each score, is represented by the color scheme.

### Datasets

All cell type labels and metadata were obtained from original publications unless specifically stated otherwise below.

#### Brain data

The mouse brain dataset is a collection of four publicly available scRNA-seq mouse brain studies^1,33–35^, for which additional information on cerebral regions was provided. We obtained the raw count matrix from Rosenberg et al.^34^ under GEO accession ID GSE110823, the annotated count matrix from Zeisel et al.^35^ from http://mousebrain.org (file name L5_all.loom, downloaded on 9 September 2019) and count matrices per cell type from Saunders et al.^33^ from http://dropviz.org (DGE by region section, downloaded on 30 August 2019). Data from mouse brain tissue sorted by flow cytometry (myeloid and non-myeloid cells, including the annotation file annotations_FACS.CSV) from TM were obtained from https://figshare.com (retrieved 14 February 2019). We harmonized cluster labels via fuzzy string matching and attempted to preserve the original annotation as far as possible. Specifically, we annotated ten major cell types (neuron, astrocyte, oligodendrocyte, oligodendrocyte precursor cell, endothelial cell, brain pericyte, ependymal cell, olfactory ensheathing cell, macrophage and microglia). In the case of Saunders et al.^33^, we facilitated the additional annotation data table for 585 reported cell types (annotation.BrainCellAtlasSaundersversion2018.04.01.TXT retrieved from http://dropviz.org on 30 August 2019. Among these, some cell types were annotated as ‘endothelial tip’, ‘endothelial stalk’ and ‘mural’. We examined the subset of the Saunders et al.^33^ dataset as follows: we used Louvain clustering (default resolution parameter, 1.0) to cluster, followed by gene expression profiling via the rankgenesgroups function in scanpy. Using marker gene expression, we assigned microglia (*C1qa*), oligodendrocytes (*Plp1*), astrocytes (*Gfap*, *Clu*) and endothelial cells (*Flt1*) to the subset. Finally, we applied scran^73^ normalization and log (counts + 1) to transform count matrices. In total, the dataset consists of 978,734 cells.

#### Pancreas

Five publicly available pancreatic islet datasets^74–78^, with a total of 15,681 cells in raw count matrix format were obtained from the Scanorama^42^ dataset, which has already assigned its cell types using batch-corrected gene expression by Scanorama. The Scanorama dataset was downloaded from http://scanorama.csail.mit.edu/data.tar.gz. In the preprocessing step, raw count datasets were normalized and log transformed by scanpy preprocessing methods. Preprocessed data were used directly for the pipeline of scArches. One thousand HVGs were selected for training the model.

#### The human cell landscape

The HCL dataset was obtained from https://figshare.com/articles/HCL_DGE_Data/7235471. Raw count matrix data for all tissues were aggregated. A total of 277,909 cells were selected and processed using the scanpy Python package. Data were normalized using size factor normalization such that every cell had 10,000 counts and then log transformed. Finally, 5,000 HVGs were selected as per their average expression and dispersion. We used processed data directly for training scArches at the pre-training phase.

#### The mouse cell atlas

The mouse cell atlas dataset was obtained from https://figshare.com/articles/HCL_DGE_Data/7235471. Raw count matrix data for all tissues were aggregated together. A total of 150,126 cells were selected and processed using the scanpy Python package. Homologous genes were selected using BioMart 100 before merging with HCL data. Data were normalized together with HCL as explained before.

#### Immune data

The immune dataset consists of ten human samples from two different tissues: bone marrow and peripheral blood. Data from bone marrow samples were retrieved from Oetjen et al.^36^, while data from peripheral blood samples were obtained from 10x Genomics (https://support.10xgenomics.com/single-cell-gene-expression/datasets/3.0.0/pbmc_10k_v3), Freytag et al.^37^, Sun et al.^38^ and Villani et al.^79^. Details on the retrieval location of datasets, the different protocols used and ways in which samples were chosen for analysis can be found in Luecken et al.^7^. We performed quality control separately for each sample but adopted a common strategy for normalization: all samples for which count data were available were individually normalized by scran pooling^73^. This excludes data from Villani et al.^79^, which included only TPM values. All datasets were log+1 transformed in scanpy^80^. Cell type labels were harmonized starting from existing annotations (Oetjen et al.^36^) to create a consistent set of cell identities. Well-known markers of cell types were collected and used to extend annotation to samples for which they were not previously available. When necessary, subclustering was performed to derive more precise labeling. Finally, cell populations were removed if no label could be assigned. Four thousand HVGs were selected for training.

#### Endocrine pancreas

The raw dataset of pancreatic endocrinogenesis (*n* = 22,163)^45^ is available at the GEO under accession number GSE132188. We considered a subset of 2,000 HVGs for training. Cell type labels were obtained from an adata object provided by the authors of scVelo^46^.

#### CITE-seq

We obtained three publicly available datasets from 10x Genomics, already curated and preprocessed as described in the totalVI study^32^. These data include ‘10k PBMCs from a Healthy Donor—Gene Expression and Cell Surface Protein’ (PBMC, 10k (CITE-seq)^81^), ‘5k PBMCs from a healthy donor with cell surface proteins (v3 chemistry)’ (PBMC, 5k (CITE-seq)^82^) and ‘10k PBMCs from a Healthy Donor (v3 chemistry)’ (PBMC, 10k (RNA-seq)^57,83,84^). Reference data included 14 proteins, and 4,000 HVGs were selected for training.

#### COVID-19

The COVID-19 dataset along with its metadata was downloaded from https://www.ncbi.nlm.nih.gov/geo/query/acc.cgi?acc=GSE1459261 and https://github.com/zhangzlab/covid_balf. The dataset that was used in this paper includes *n* = 62,469 cells. Data from lungs^52–54^, PBMCs^37–39^ and bone marrow^36^ were later merged with those from COVID-19 samples. Data were normalized using scanpy, and 2,000 HVGs were selected for training the model. Cell type labels were obtained from the original study.

#### Tabula Muris Senis

The TM Senis dataset with GEO accession number GSE132042 is publicly available at https://figshare.com/projects/Tabula_Muris_Senis/64982. The dataset contains 356,213 cells with cell type, tissue and method annotation. We normalized the data using size factor normalization with 10,000 counts for each cell. Next, we log+1 transformed the dataset and selected 5,000 HVGs according to their average expression and dispersion. All preprocessing steps were carried out using the scanpy Python package. In this study, we used a combination of sequencing technology and time point as batch covariates.

### Benchmarks

#### Full integration methods

We ran PCA with 20 principal components on the final results from Seurat, Scanorama and mnnCorrect to be comparable (similar approach as described in ref. ^31^) when computing metrics to deep learning models, which had a latent representation of size 10–20.

- Harmony: we used the Harmony Matrix function from the Harmony package. We provided the function with a PCA matrix with 20 principal components on the gene expression matrix.
- Scanorama: we used the correct_scanpy function from the Scanorama package with default parameters.
- Seurat: we applied Seurat as in the walkthrough (https://satijalab.org/seurat/v3.1/integration.html) with default parameters.
- Liger: we used the Liger method as in the walkthrough (https://github.com/welch-lab/liger/blob/master/vignettes/walkthrough_pbmc.pdf). We used *k* = 20, *λ* = 5 and resolution = 0.4 with other default parameters. We only scaled data as we had already preprocessed data.
- Conos: we followed the Conos tutorial at https://htmlpreview.github.io/?https://raw.githubusercontent.com/kharchenkolab/conos/master/doc/walkthrough.html. Unlike the tutorial, we used our own preprocessed data for better comparisons. We used PCA space with parameters *k* = 30, k.self = 5, ncomps = 30, matching.method = ’mNN’ and metric = ’angular’ to build the graph. We set the resolution to 1 to find communities. Finally, we saved the corrected pseudo-PCA space with 20 components.
- mnnCorrect: we used the mnnCorrect function from the scran package with default parameters.

#### Cell type-classification methods

- Seurat: we followed the walkthrough (https://satijalab.org/seurat/v3.1/integration.html) and used reciprocal PCA for dimension reduction. As described in the original publication^48^, we examined projection scores and assigned cells with the lowest 20% of values to be ‘unknown’.
- SVM: we fitted an SVM model from the scikit-learn library to the reference data and classified query cells. We assigned cells with uncertainty probability greater than 0.7 as ‘unknown’.
- Logistic regression: we fitted logistic regression from the scikit-learn library to the reference data and predicted query labels.

All these methods were tested on a machine with one eight-core Intel i7-9700KQ CPU addressing 32 GB RAM and one Nvidia GTX 1080 ti (12 GB) addressing 12 GB VRAM.

### Model output

Throughout this paper, all low-dimensional representations were obtained using the latent space of scArches models. The output of scArches models will be confounded with condition variables not fit for data-integration applications but best for imputation or denoising scenarios.

### Cell type annotation

To classify labels for the query dataset, we trained a weighted kNN classifier on the latent-space representation of the reference dataset. For each query cell *c*, we extracted its kNNs (*N*_*c*_). We computed the standard deviation of the nearest distances:

$${\textrm{s.d.}}_{c,N_c} = \sqrt {\frac{{\mathop {\sum}\nolimits_{n \in N_c} {({\textrm{dist}}(c,n))^2} }}{k}},$$

where dist(*c*, *n*) is the Euclidean distance of the query cell *c* and its neighbors *n* in the latent space. Next, we applied the Gaussian kernel to distances using

$$D_{c,n,N_c} = e^{ - \frac{{\textrm{dist}(c,n)}}{{(2/{\textrm{s.d.}}_{c,N_c})^2}}}.$$

Next, we computed the probability of assigning each label *y* to the query cell *c* by normalizing across all adjusted distances using

$$p(Y = y|X = c,N_c) = \frac{{\mathop {\sum}\nolimits_{i \in N_c} {I(y^{(i)} = y) \cdot D_{c,n_i,N_c}} }}{{\mathop {\sum}\nolimits_{j \in N_c} {D_{c,n_j,N_c}} }},$$

where *y*^(*i*)^ is the label of *i*th nearest neighbor and *I* is the indicator function. Finally, we calculated the uncertainty *u* for each cell *c* in the query dataset using its set of closest neighbors in the reference dataset (*N*_*c*_). We defined the uncertainty $u_{c,y,N_c}$ for a query cell *c* with label *y* and *N*_*c*_ as its set of nearest neighbors as

$$u_{c,y,N_c} = 1 - p(Y = y|X = c,N_c).$$

We reported cells with more than 50% uncertainty as unknown to detect out-of-distribution cells with new labels, which do not exist in the training data. Therefore, we labeled each cell *c* in the query dataset as follows:

$$\begin{array}{l}\hat y_c^\prime = {\textrm{argmin}}_y\,u_{c,y,N_c}\\ \hat{y}_c = \left\{ \begin{array}{lr} \hat{y}^\prime_c & {\mathrm{if}}\ u_{c, \hat{y}^\prime_c, N_c} {\le} 0.5 \\ {\mathrm{unknown}} & {\mathrm{o.w.}}\end{array} \right\}\end{array}$$

#### Protein imputation

For scArches totalVI, missing proteins for RNA-seq-only data were imputed by conditioning query cells as being in the other batches in the reference with protein data. It is possible to impute based on a specific batch or average across all batches. In the example in the paper, the average version was used.

### Reporting Summary

Further information on research design is available in the Nature Research Reporting Summary linked to this article.

## Online content

Any methods, additional references, Nature Research reporting summaries, source data, extended data, supplementary information, acknowledgements, peer review information; details of author contributions and competing interests; and statements of data and code availability are available at 10.1038/s41587-021-01001-7.

## Supplementary information

Supplementary Information Supplementary Notes 1–3, Tables 1–7 and Figs. 1–28.

Reporting Summary

## Data Availability

All datasets used in the paper are public, referenced and downloadable at https://github.com/theislab/scarches-reproducibility.

## Code availability

Software is available at https://github.com/theislab/scarches. The code to reproduce the results is available at https://github.com/theislab/scarches-reproducibility.
