# Supplementary material for: Mapping single-cell data to reference atlases by transfer learning
Source: Nat Biotechnol. 2021 Aug 30;40(1):121–30. doi: 10.1038/s41587-021-01001-7 (PMC8763644; doi:10.1038/s41587-021-01001-7)
Supplement: Supplementary file 1 — Supplementary Notes 1–3, Tables 1–7 and Figs. 1–28. [file 41587_2021_1001_MOESM1_ESM.pdf]

nature portfolio

https://doi.org/10.1038/s41587-021-01001-7

Supplementary information

Mapping single-cell data to reference atlases by transfer learning

In the format provided by the authors and unedited

1 Supplementary Note 1

Atlas-level integration

Using atlas type and sequencing technique as batch labels, our model successfully groups similar tissues from different atlases while preserving the heterogeneity within each tissue (**Supplementary Figure 18a**). Illustratively, we further examined two major brain cell clusters after integration. scArches successfully aligned the query microglia cells to the myeloid brain cell cluster in the reference while non-immune glial cells such as astrocytes and oligodendrocytes were correctly integrated to the non-myeloid cluster (**Supplementary Figure 18b**). Thus, transferring weights from the TS reference model to co-embed MCA data enabled the integration of different atlases to overcome batch effects from different laboratories, technologies, and ages.

In addition to integrating studies from an organism, it is instructive to assess the similarity of cell types across species. Illustratively, we trained a reference model based on the recently published Human Cell Landscape (HCL) [1] comprised of 249, 845 cells across 63 human tissues. After architecture surgery, we aligned the MCA (n=122, 944) into the reference human cells (**Supplementary Figure 18c**). Given the abundance of species-specific cell types and species-specific functions of particular cell types, we do not expect all cell types to overlap across species. Yet, we find that specifically similar immune cell types, such as neutrophils and macrophages, were clustered together across species while species-specific cell types were placed separately (**Supplementary Figure 18d**). These observations agreed with the analysis by aggregating cells from the same cluster into one cell (pseudo-cell) from the original publication [1], confirming a high similarity of the immune cells and other major cell type across both species. Overall, the strong regularization of the transfer from reference via scArches allows the integration to overcome the strong species biological effect and focus on the gene expression similarity across major mammalian cell types (**Supplementary Figures 19, 20, 21**); we thus believe that the resulting resource can offer a basis for cross-species analysis of cell-type identity.

2 Supplementary Note 2

scArches preserves biological variability and variability from ambient RNA during reference construction and query mapping

We have demonstrated the successful contextualization of diverse cellular populations from patients with COVID-19 with cells obtained from healthy volunteers and normal donor tissues. Yet, while tissue-resident alveolar macrophages (TRAMs) from query control (healthy volunteers) and reference (normal donor lung) are placed within the same broad cluster, they are not fully mixed together. Thus, we asked from where this separation originates. To investigate this, we performed Leiden clustering of the integrated macrophages (**Supplementary Figure 23a-d**). While most cell atlases in the reference were dominated by alveolar macrophages (*FABP4+, MARCO+*; **Supplementary Figure 23f**) some cell atlases (specifically HCL[1] and Madissoon datasets[2]) contained additional populations of monocytes (*FCN1+, S100A8+, SOD2+*; **Supplementary Figure 23g**) and monocyte-derived macrophages (*CCL2+, SPP1+*; **Supplementary Figure 23h**). Notably, scArches also preserved biological variability and some technical variability (resulting from different composition of ambient RNA) within *bona fide* alveolar macrophages (*FABP4+, MARCO+*). For example, in comparison to other samples, macrophages from Travaglini et al.[3] were characterized by increased expression of *IL1RN* and *IL1B* (**Supplementary Figure 23i**), suggesting their activation. This is not surprising, as these samples were obtained not from healthy subjects, but rather from patients with lung cancer. Similarly, the scArches reference model still preserved some technical variability: alveolar macrophages from Reyfman et al.[4] were characterized by increased levels of transcripts encoding surfactant proteins (**Supplementary Figure 23j**). This can be explained by the protocol used by Refyman and colleagues, which favors isolation of surfactant-producing alveolar epithelial type 2 cells which contributed to higher levels of ambient RNA. This suggests reference model construction could have been improved with further ambient RNA removal. Finally, samples from control query (Liao et al.[5]), which were obtained via bronchoalveolar lavage procedure from

healthy volunteers, rather than enzymatic tissue digestion of explanted lungs, were characterized by increased expression of IFN-responsive genes *IFIT2* and *IFIT3* (**Supplementary Figure 23k**). Enrichment for alveolar macrophages with an interferon-responsive gene signature in bronchoalveolar lavage fluid was recently reported by Mould and colleagues [6], suggesting that IFN-responsive alveolar macrophages are more likely to detach.

We further analysed the separation of different CD8+ T cells populations (see **Supplementary Figure 26a-d**). Leiden clustering demonstrated that naive CD8+ T cells (*CCL5*-) originating from bone marrow and peripheral blood formed a separate cluster from effector cytotoxic CD8+ T cells originating from lung tissue and bronchoalveolar lavage (*CCL5*+, *GZMA*+, *GZMH*+, see **Supplementary Figure 26e**). Moreover, CD8+ T cells from patients with COVID-19 were characterized by the expression of interferon-responsive genes (*ISG15*, *MX1*, and others), which was recently reported as a specific feature of SARS-CoV-2 pneumonia[7].

Together, our analysis suggest that scArches preserves biological variability both within reference and query.

3 Supplementary Note 3

The importance of choosing the right reference

To contextualize query data from COVID-19 patients, we generated a healthy reference dataset from lung, PBMCs, and bone marrow immune and epithelial cells. These tissues were chosen as they contain similar cell types and thus may help to contextualize the cells that we find in the COVID-19 patient data. Given that the focus of this paper is not optimal reference building, we merged these datasets using a previously well-evaluated model class (NB CVAE from scVI [8]) [9]. However, evaluating the results of mapping the COVID-19 query data into the reference model is complicated by a lack of harmonized cell type labels (e.g., some datasets have cells labeled T cells, while others separate into T cell subtypes) and the quality of the reference. These aspects are particularly complicated for the data from the Human Cell Landscape [1], which have coarse labels and do not integrate well with the rest of the data (**Supplementary Figures 24,27**). For example, cells with the label "T" from Liao et al seem to be CD4+ T cells (see violin plot in **Supplementary Figure 27**), but have the same label as T cells without CD4 or CD8 expression from the HCL dataset (**Supplementary Figure 27**). Indeed, our integration shows that query "T" cells map to CD4+ T cells, while the "T" cells form the HCL separate by tissue and fail to integrate into the reference. Without the effect of the HCL dataset, T cells clearly separate into CD4+ and CD8+ expressing cells across tissues and unharmonized labels. Overall, our simple reference contained sufficient relevant cells to cache the effect of the poor HCL reference. However, this may not always be the case for references used to contextualize query data. Thus, choosing the appropriate reference is of critical importance for reference mapping using scArches.

References

[1] Han, X. et al. Construction of a human cell landscape at single-cell level. Nature 1–9 (2020).

[2] Madissoon, E. et al. scrna-seq assessment of the human lung, spleen, and esophagus tissue stability after cold preservation. Genome Biology **21**, 1–16 (2020).

[3] Travaglini, K. J. et al. A molecular cell atlas of the human lung from single cell RNA sequencing. bioRxiv (2020).

[4] Single-Cell Transcriptomic Analysis of Human Lung Provides Insights into the Pathobiology of Pulmonary Fibrosis | American Journal of Respiratory and Critical Care Medicine.

[5] Liao, M. et al. Single-cell landscape of bronchoalveolar immune cells in patients with COVID-19. Nature Medicine 1–3 (2020).

[6] Mould, K. J. et al. Airspace Macrophages and Monocytes Exist in Transcriptionally Distinct Subsets in Healthy Adults. American Journal of Respiratory and Critical Care Medicine (2020). Publisher: American Thoracic Society - AJRCCM.

[7] Grant, R. A. et al. Circuits between infected macrophages and T cells in SARS-CoV-2 pneumonia. Nature 1–10 (2021). Publisher: Nature Publishing Group.

[8] Lopez, R., Regier, J., Jordan, M. I. & Yosef, N. Information constraints on auto-encoding variational bayes. In Advances in Neural Information Processing Systems, 6114–6125 (2018).

[9] Luecken, M. D. et al. Benchmarking atlas-level data integration in single-cell genomics. bioRxiv 2020.05.22.111161 (2020).

4 Hyperparameters

**Experiment**	**condition label**
Pancreas	Batch (datasets)
Brain	Batch (datasets)
Immune	Batch (datasets)
TM	Technology_Age (e.g. Droplet_3months)
TM-TS-MCA	Technology_Age
Cross-species (MCA, HCL)	Stage(age)_Species (e.g. Adult _Human)
CITEseq multi-modal	Batch (datasets)
COVID-19	Batch (datasets)

Supplementary Table 1 | The label type used for each experiment as conditions for scArches CVAE models.

Name	Operation	NoF/Kernel Dim.	Dropout	LN	Activation	Input
**Inputs**						
data	-	#Genes	×	×	-	-
conditions	-	#Conditions	×	×	-	-
library	Sum of counts	1	×	×	-	-
**Encoder**						
Layer_1	FC	128	0.1	√	ReLU	[data, **study labels]**
Layer_2	FC	128	0.1	√	ReLU	Layer_1
mean	FC	10	×	×	Linear	Layer_2
var	FC	10	×	×	Linear	Layer_2
latent	Multivariate Normal	10	×	×	-	[mean, var]
**Decoder**						
Layer_1	FC	128	0.1	√	ReLU	[latent, **study labels]**
Layer_2	FC	128	0.1	√	ReLU	Layer_1
scale	FC	#Genes	×	×	Softmax	Layer_2
mean	Multiplication	#Genes	×	×	-	[library, scale]
dropout	FC	#Genes	×	×	Linear	Layer_2
dispersion	Parameter	#Genes	×	×	-	-
**Hyperparameters**						
Loss	ZINB					
Optimizer	Adam					
Learning Rate	0.001					
ϵ	0.01					
Weight Decay	0,000001					
Batch Size	128					
# of Epochs	Early Stopping on ELBO	max. 500 Epochs				
α (KL term weight)	Alpha annealing to 1	400 Epochs				

Supplementary Table 2 | scArches scVI detailed architecture. We used the same architecture for all the examples in the paper.

Name	Operation	NoF/Kernel Dim.	Dropout	LN	Activation	Input
**Inputs**						
data	-	#Genes	×	×	-	-
conditions	-	#Conditions	×	×	-	-
cell-types	-	#Cell-types	×	×	-	-
library	Sum of counts	1	×	×	-	-
**Encoder**						
Layer_1	FC	128	0.1	√	ReLU	[data, **study labels]**
Layer_2	FC	128	0.1	√	ReLU	Layer_1
mean	FC	10	×	×	Linear	Layer_2
var	FC	10	×	×	Linear	Layer_2
latent	Multivariate Normal	10	×	×	-	[mean, var]
**Decoder**						
Layer_1	FC	128	0.1	√	ReLU	[latent, **study labels]**
Layer_2	FC	128	0.1	√	ReLU	Layer_1
scale	FC	#Genes	×	×	Softmax	Layer_2
mean	Multiplication	#Genes	×	×	-	[library, scale]
dropout	FC	#Genes	×	×	Linear	Layer_2
dispersion	Parameter	#Genes	×	×	-	-
**KL shrinkage**						
Layer_1	FC	128	0.1	√	ReLU	[latent, cell-types]
Layer_2	FC	128	0.1	√	ReLU	[Layer_1, cell-types]
kl_mean	FC	10	×	×	Linear	Layer_2
kl_var	FC	10	×	×	Linear	Layer_2
kl_latent	Multivariate Normal	10	×	×	-	[kl_mean, kl_var]
Layer_3	FC	128	0.1	√	ReLU	[kl_latent, cell-types]
Layer_4	FC	128	0.1	√	ReLU	[Layer_3, cell-types]
kl_mean_2	FC	10	×	×	Linear	Layer_4
kl_var_2	FC	10	×	×	Linear	Layer_4
**Classifier**						
Layer_1	FC	128	0.1	√	ReLU	latent
Layer_2	FC	128	0.1	√	ReLU	Layer_1
output	FC	#Cell-types	×	×	Softmax	Layer_2
**Hyperparameters**						
Loss	ZINB					
Optimizer	Adam					
Learning Rate	0.001					
ϵ	0.01					
Weight Decay	0,000001					
Batch Size	128					
# of Epochs	Early Stopping on ELBO	max. 500 Epochs				
α (KL term weight)	Alpha annealing to 1	400 Epochs				
Classifier LR	0.005					
Classifier Factor	50					
Classifier Epochs	1					

Supplementary Table 3 | scArches scANVI detailed architecture. We used the same architecture for all the examples in the paper.

Name	Operation	NoF/Kernel Dim.	Dropout	LN	Activation	Input
**Inputs**						
data	-	#Genes	×	×	-	-
protein	-	#Proteins	×	×	-	-
conditions	-	#Conditions	×	×	-	-
cell-types	-	#Cell-types	×	×	-	-
library	Sum of gene counts	1	×	×	-	-
**Encoder**						
Layer_1	FC	256	0.2	✓	ReLU	[data, protein, **study labels**]
Layer_2	FC	256	0.2	✓	ReLU	[Layer_1, **study labels**]
mean	FC	20	×	×	Linear	Layer_2
var	FC	20	×	×	Linear	Layer_2
latent	Multivariate Normal	20	×	×	-	[mean, var]
**RNA Decoder**						
Layer_1	FC	256	0.2	✓	ReLU	[latent, **study labels**]
scale	FC	#Proteins	×	×	Softmax	[Layer_1, latent, **study labels**]
mean	Multiplication	#Genes	×	×	-	[library, scale]
dropout	FC	#Genes	×	×	Linear	[sigmoid_decoder, latent, **study labels**]
gene_dispersion	Parameter	#Genes	×	×	-	-
**Protein Decoder**						
background	FC	256	0.2	✓	ReLU	[latent, **study labels**]
background_alpha	FC	#Proteins	×	×	Linear	[background, latent, **study labels**]
background_beta	FC	#Proteins	×	×	Linear	[background, latent, **study labels**]
background_mean	Multivariate Normal	#Proteins	×	×	-	[background_alpha, background_beta]
foreground	FC	256	0.2	✓	ReLU	[latent, **study labels**]
foreground_scale	FC	#Proteins	×	×	ReLU	[foreground, latent, **study labels**]
foreground_rate	Multiplication	#Proteins	×	×	-	[background_mean, foreground_scale]
sigmoid_decoder	FC	256	0.2	✓	ReLU	[latent, **study labels**]
mixing	FC	#Proteins	×	×	Linear	[sigmoid_decoder, latent, **study labels**]
protein_dispersion	Parameter	#Proteins	×	×	-	-
**Hyperparameters**						
Loss	NB					
Optimizer	Adam					
Learning Rate	0.004					
ϵ	0.01					
Weight Decay	0,000001					
Batch Size	256					
# of Epochs	400					
α (KL term weight)	Alpha annealing to 1	8000 Epochs				

Supplementary Table 4 | scArches totalVI detailed architecture. We used the same architecture for all the examples in the paper.

Name	Operation	NoF/Kernel Dim.	Dropout	LN	Activation	Input
**Inputs**						
data	-	#Genes	×	×	-	-
conditions	-	#Conditions	×	×	-	-
**Encoder**						
Layer_1	FC	128	0.1	✓	ReLU	[data, **study labels**]
Layer_2	FC	20	0.1	✓	ReLU	Layer_1
mean	FC	10	×	×	Linear	Layer_2
var	FC	10	×	×	Linear	Layer_2
latent	Multivariate Normal	10	×	×	-	[mean, var]
**Decoder**						
Layer_1	FC	20	0.1	✓	ReLU	[latent, **study labels**]
Layer_2	FC	128	0.1	✓	ReLU	Layer_1
mean	Multiplication	#Genes	×	×	-	[library, scale]
**Hyperparameters**						
Loss	MSE					
Optimizer	Adam					
Learning Rate	0.001					
ϵ	0.01					
Batch Size	128					
# of Epochs	Early Stopping on ELBO	max. 500 Epochs				
α (KL term weight)	0.1 (Immune), 5.0 (Pancreas), 0.2 (Mouse Brain)					
β (MMD term weight)	0.1 (Immune), 2.0 (Pancreas), 0.15 (Mouse Brain)					

Supplementary Table 5 | scArches trVAE detailed architecture. We used the same architecture for all the examples in the paper.

Name	Operation	NoF/Kernel Dim.	Dropout	LN	Activation	Input
**Inputs**						
data	-	#Genes	×	×	-	-
conditions	-	#Conditions	×	×	-	-
**Encoder**						
Layer_1	FC	128	0.1	√	ReLU	[data, **study labels**]
Layer_2	FC	20	0.1	√	ReLU	Layer_1
mean	FC	10	×	×	Linear	Layer_2
var	FC	10	×	×	Linear	Layer_2
latent	Multivariate Normal	10	×	×	-	[mean, var]
**Decoder**						
Layer_1	FC	20	0.1	√	ReLU	[latent, **study labels**]
Layer_2	FC	128	0.1	√	ReLU	Layer_1
mean	Multiplication	#Genes	×	×	-	[library, scale]
**Hyperparameters**						
Loss	MSE					
Optimizer	Adam					
Learning Rate	0.001					
ϵ	0.01					
Batch Size	128					
# of Epochs	Early Stopping on ELBO	max. 500 Epochs				
α KL term weight)	0.05 (Immune), 5.0 (Pancreas), 0.1 (Mouse Brain)					

Supplementary Table 6 | scArches CVAE (MSE) detailed architecture. We used the same architecture for all the examples in the paper.

Name	Operation	NoF/Kernel Dim.	Dropout	LN	Activation	Input
**Inputs**						
data	-	#Genes	×	×	-	-
conditions	-	#Conditions	×	×	-	-
**Encoder**						
Layer_1	FC	128	0.1	√	ReLU	[data, **study labels**]
Layer_2	FC	20	0.1	√	ReLU	Layer_1
mean	FC	10	×	×	Linear	Layer_2
var	FC	10	×	×	Linear	Layer_2
latent	Multivariate Normal	10	×	×	-	[mean, var]
**Decoder**						
Layer_1	FC	20	0.1	√	ReLU	[latent, **study labels**]
Layer_2	FC	128	0.1	√	ReLU	Layer_1
predicted count	Multiplication	#Genes	×	×	-	[library, scale]
**Hyperparameters**						
Loss	NB					
Optimizer	Adam					
Learning Rate	0.001					
ϵ	0.01					
Batch Size	128					
# of Epochs	Early Stopping on ELBO	max. 500 Epochs				
α (KL term weight)	0.1 (Immune), 1.0 (Pancreas), 0.2 (Mouse Brain)					

Supplementary Table 7 | scArches CVAE (NB) detailed architecture. We used the same architecture for all the examples in the paper.

5 Supplementary Figures

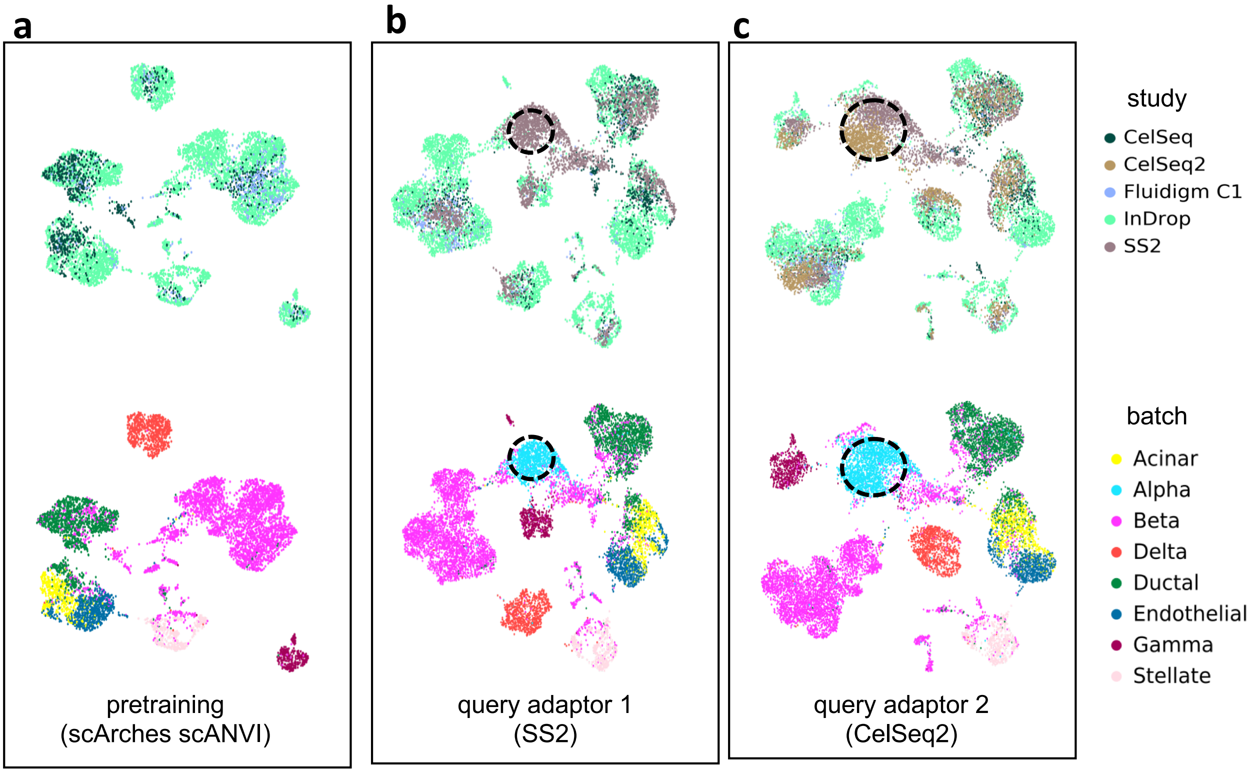

Supplementary Figure 1 | scArches scANVI reference mapping. scArches workflow for a human pancreas ($n = 15,681$) atlas. Training scArches scANVI on a reference including three human pancreas datasets (CelSeq, InDrop, Fluidigm C1). UMAP embedding for the integrated reference (**a**). Querying a new SMART-Seq2 (SS2) dataset to the integrated reference (**b**). (**c**) Updating the cell atlas with a fifth dataset (CelSeq2). The black dashed circles represent cells absent in the reference data.

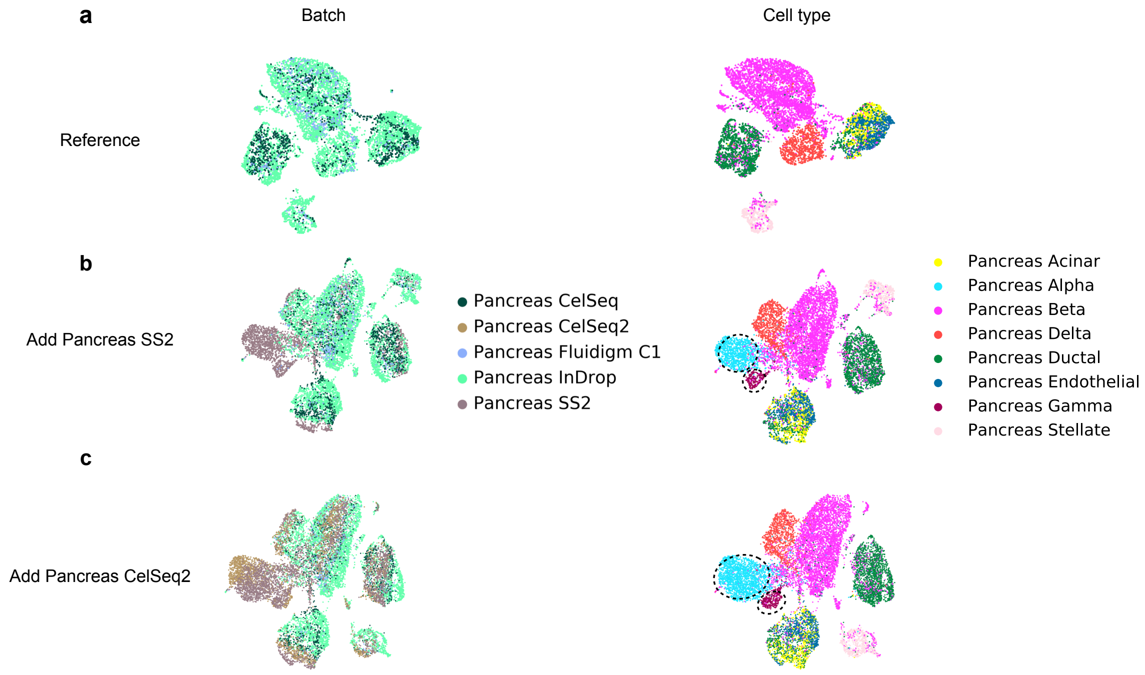

Supplementary Figure 2 | Robustness evaluation for unseen cell-types in reference. (a) UMAP representation of the pretrained scArches trVAE model with reference pancreas datasets (CelSeq, inDrop, Fluidgram C1) while Alpha and Gamma cells are absent in the data. **(b-c)** Iterative integration of two query datasets (SS2, CelSeq2) containing Alpha and Gamma cells

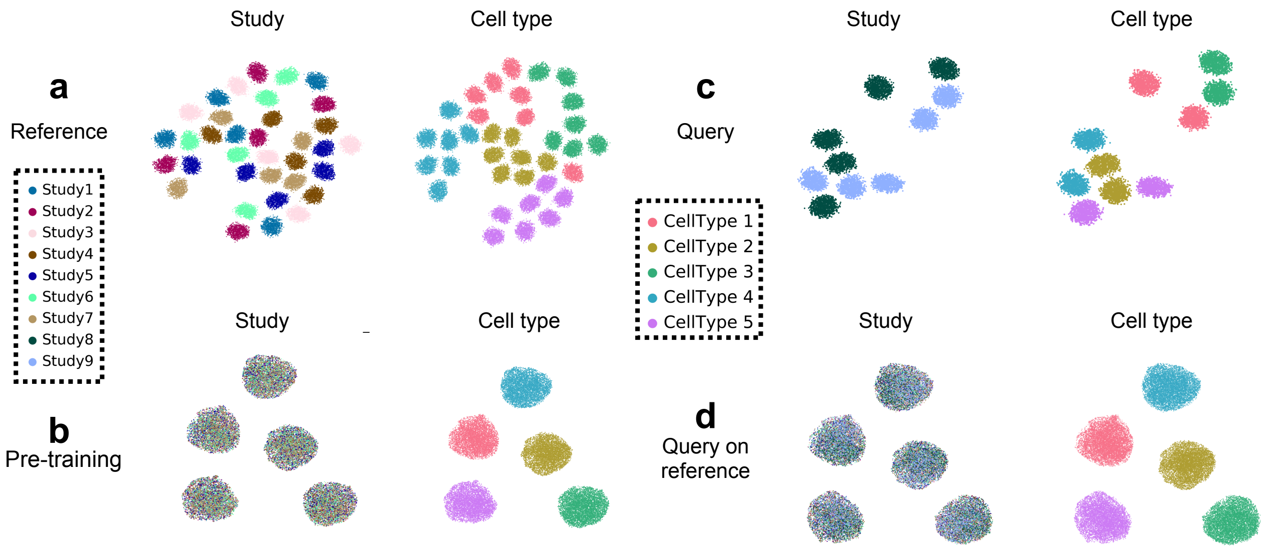

Supplementary Figure 3 | scArches applied on simulated data. (a) UMAP representation of the simulated reference data with seven batches and five different cell types. **(b)** Pretraining of the model on the reference data. **(c)** The simulated query data with two batches. **(d)** Mapping the query to the reference.

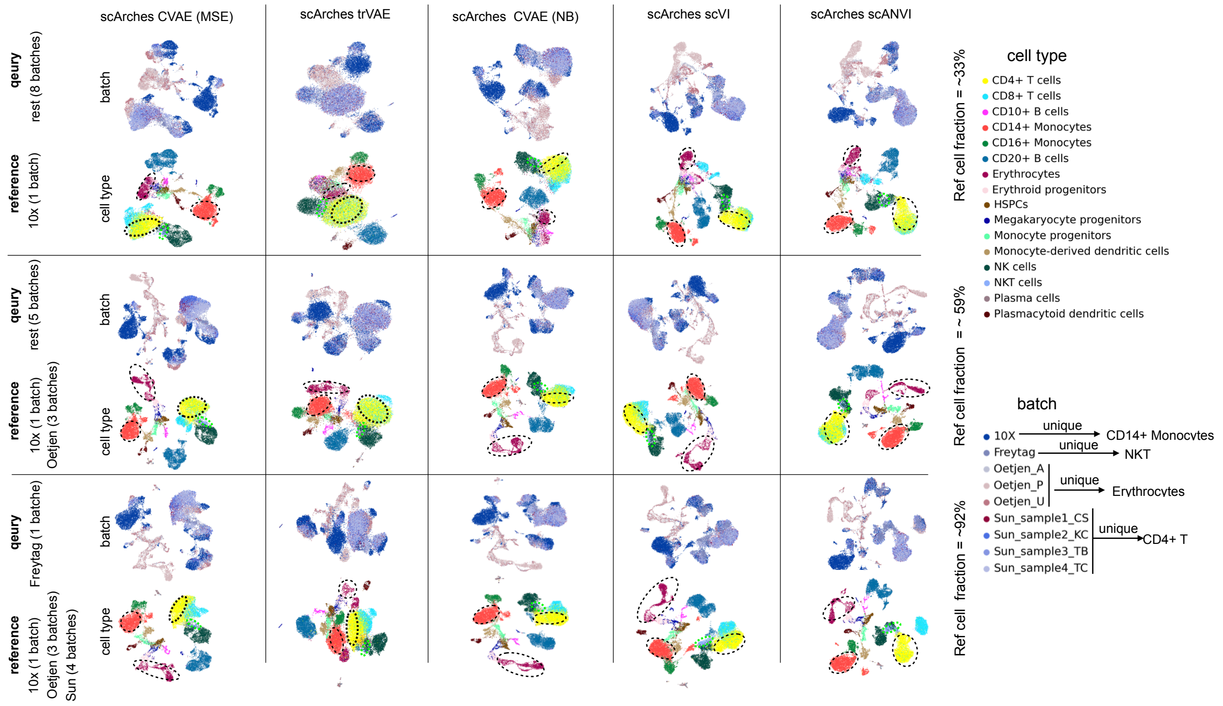

Supplementary Figure 4 | Reference fraction evaluations for Humane Immune data across various scArches base models. UMAP of the latent space for different scArches *base models* on Immune ($n = 20,522$) data while each study contained a unique cell-type not present in other studies as denoted in the batch legend. Reference ratio refers to the fraction of cells in the reference compared to all data. The studies used as reference and query are indicated at the left of each figure.

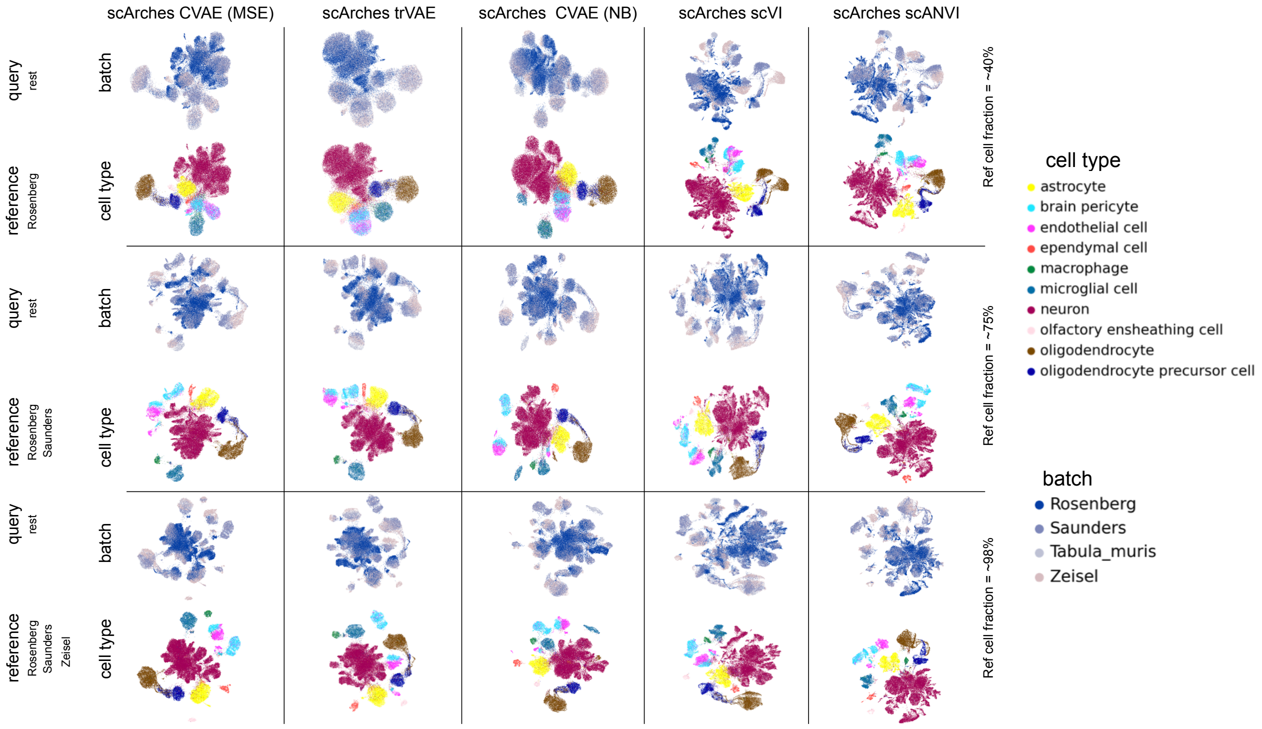

Supplementary Figure 5 | Reference fraction evaluations for Brain data across various scArches base models. UMAP of the latent space for different scArches *base models* on Brain ($n = 332,129$) dataset including four different studies. Reference ratio refers to the fraction of cells in the reference compared to all data. The studies used as reference and query are indicated at the left of each figure.

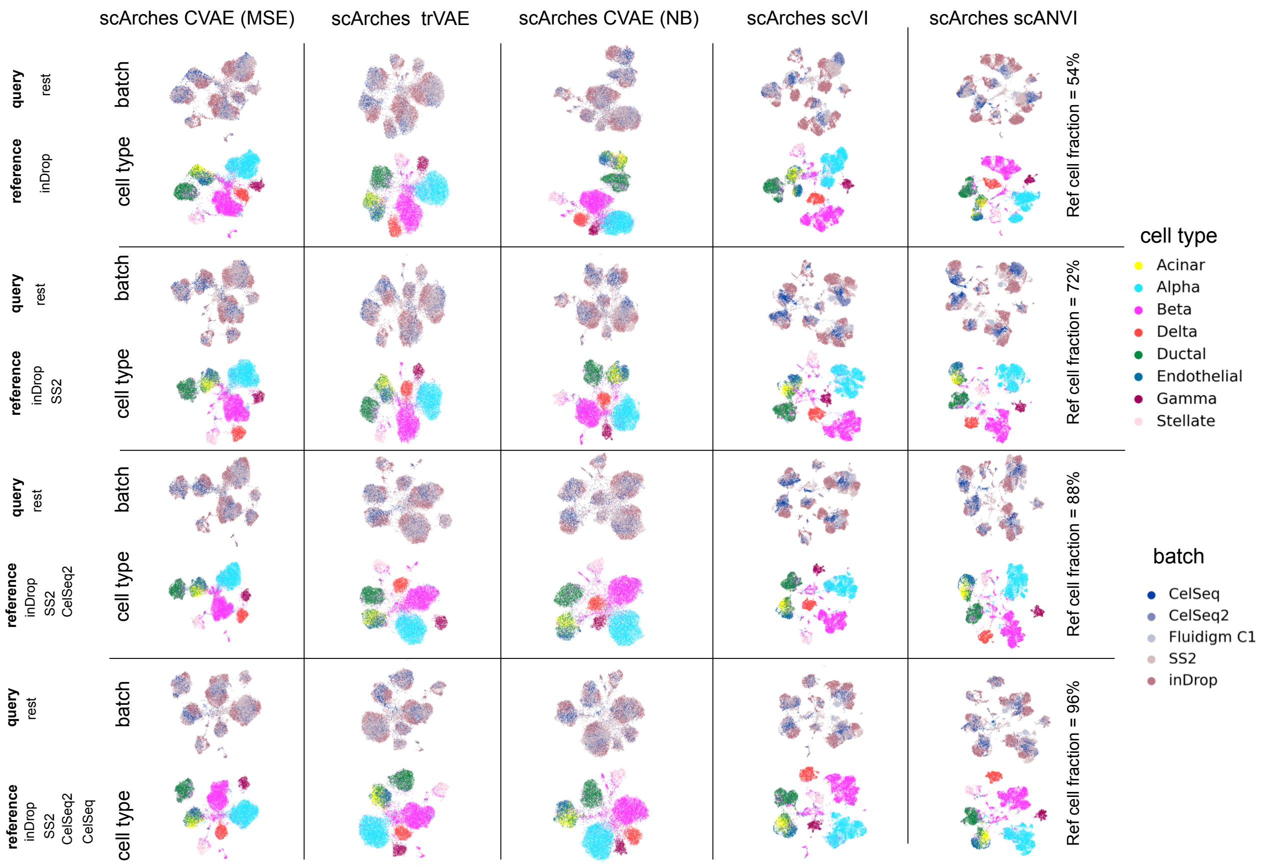

Supplementary Figure 6 | Reference fraction evaluations for Pancreas data across various scArches base models. UMAP of the latent space for different scArches *base models* on Pancreas ($n = 15,681$) dataset including five different experimental technologies. Reference ratio refers to the fraction of cells in the reference compared to all data. The studies used as reference and query are indicated at the left of each figure.

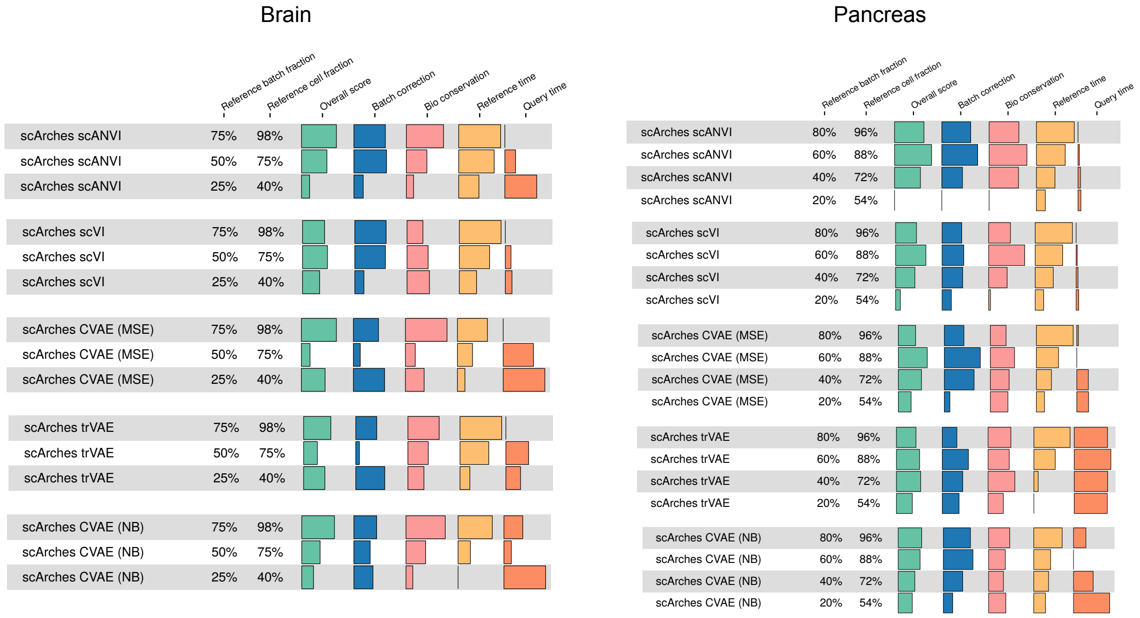

Supplementary Figure 7 | Assessment of reference fraction size on the quality of reference mapping for Brain and Pancreas data sets. The overall integration scores are obtained using weighted 40:60 mean of batch correction and bio-conservation categories, respectively (see **Methods??** for further visualization details).

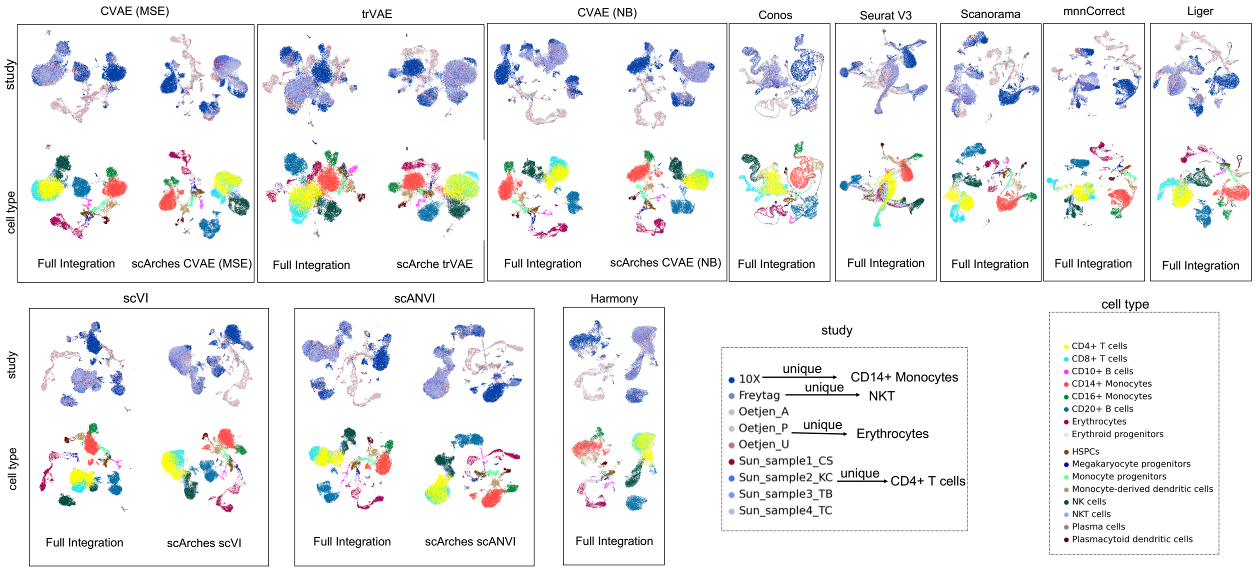

Supplementary Figure 8 | Visualisation of integrated data for scArches reference mapping against de-novo full integration methods for Human Immune data. UMAP of the latent space for different scArches *base models* and de-novo integration methods on Human Immune ($n = 20,522$) data while each study contained a unique cell-type not present in other studies as denoted in the batch legend. The scArches models were trained with a reference data including ≈2/3 of batches in the data while remaining ≈1/3 if batches were integrated as the query.

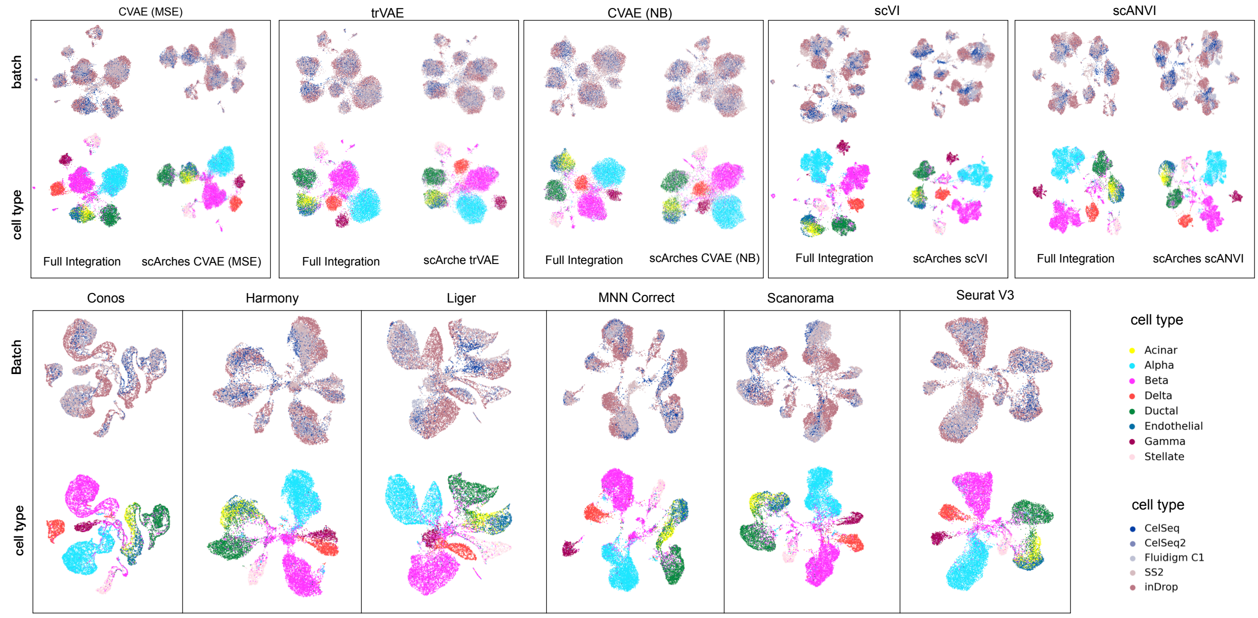

Supplementary Figure 9 | Visualisation (UMAP) of integrated data for scArches reference mapping against full integration methods for Pancreas data. UMAP of the integrated five Pancreas studies ($n = 15,681$) for scArches and de-novo integration pipelines. The scArches models were trained with a reference data including ≈2/3 of batches in the data while remaining ≈1/3 if batches were integrated as the query.

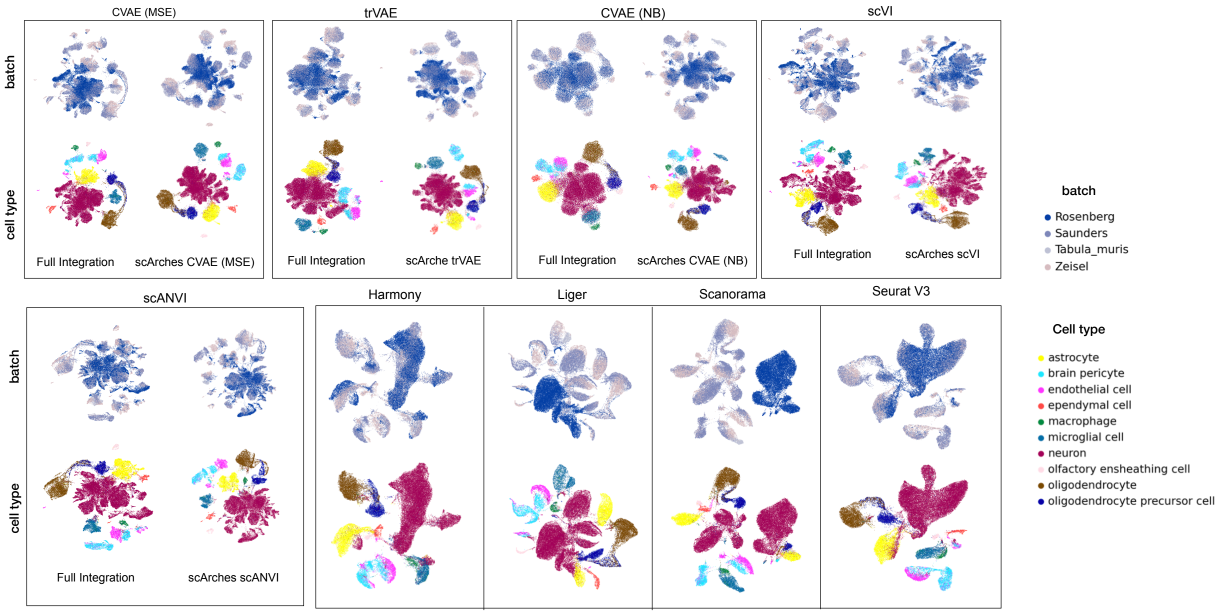

Supplementary Figure 10 | Visualisation (UMAP) of integrated data for scArches reference mapping against full integration methods for mouse Brain. UMAP of the integrated four Brain studies ($n = 332,129$) for scArches and de-novo integration pipelines. The scArches models were trained with a reference data including ≈2/3 of batches in the data while remaining ≈1/3 if batches were integrated as the query.

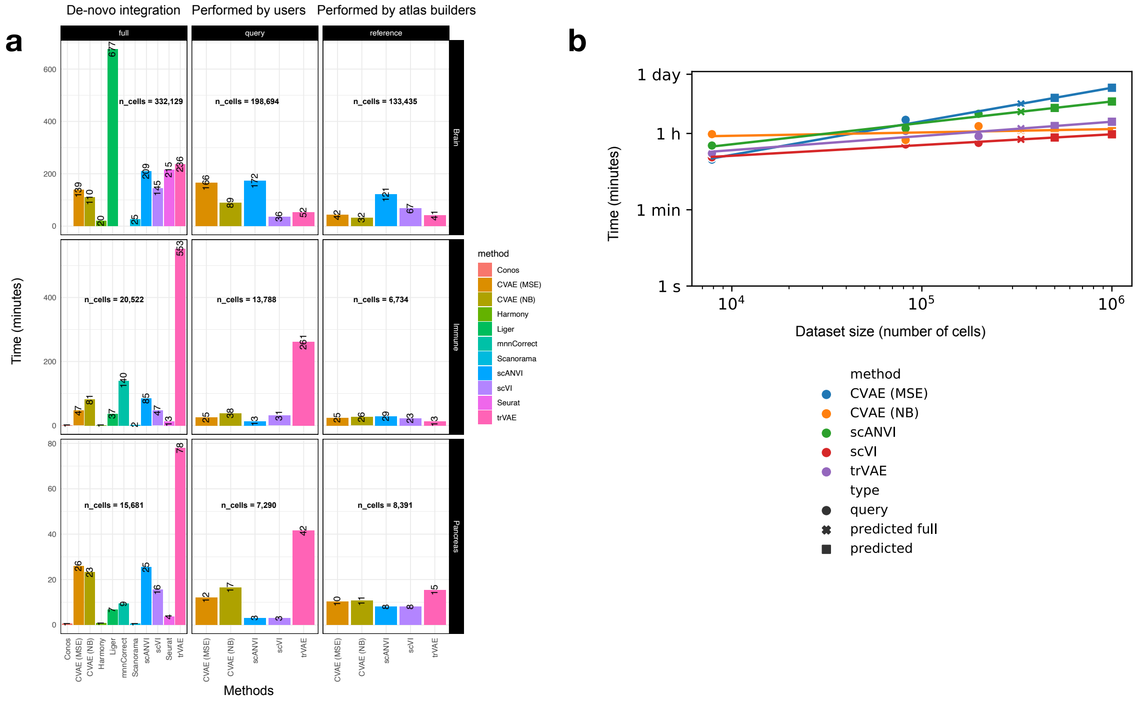

Supplementary Figure 11 | Time comparison across scArches models and full integration pipelines. (a) Comparison of user time for reference mapping using scArches versus running de-novo full integration pipe-line for Brain, Immune, and Pancreas datasets. **(b)** The estimated user time for required mapping a query datast same size as the original Brain data, 500k, and 1 million cells for different scArches base model. The estimation are for 4,000 genes as input.

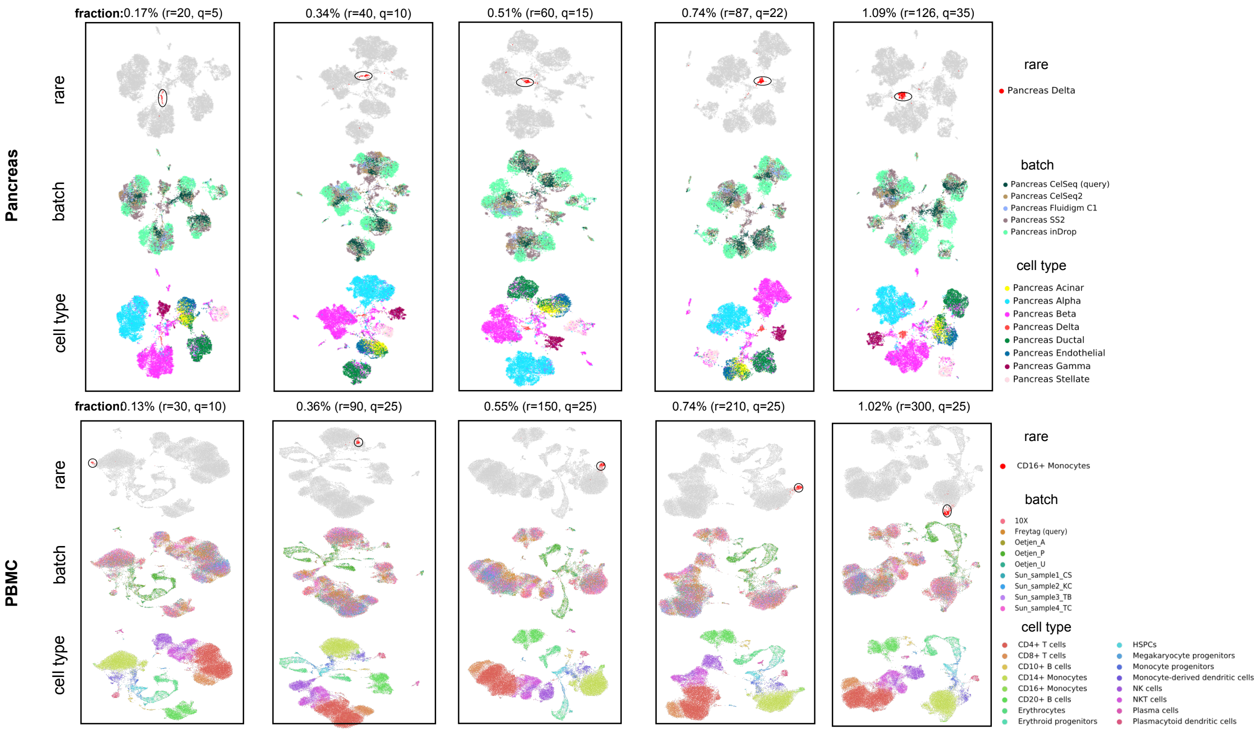

Supplementary Figure 12 | Assessing the reference mapping for rare cell types. (a-b) UMAPs for reference mapping using scArches scVI for Delta and CD16+ Monocytes across Pancreas and Immune data. The numbers above each plot denote the fraction of rare cells (query + reference) in whole data. r and q and represent number of reference and query cells for rare cell type, respectively. The dataset used as query are denoted in batch legend.

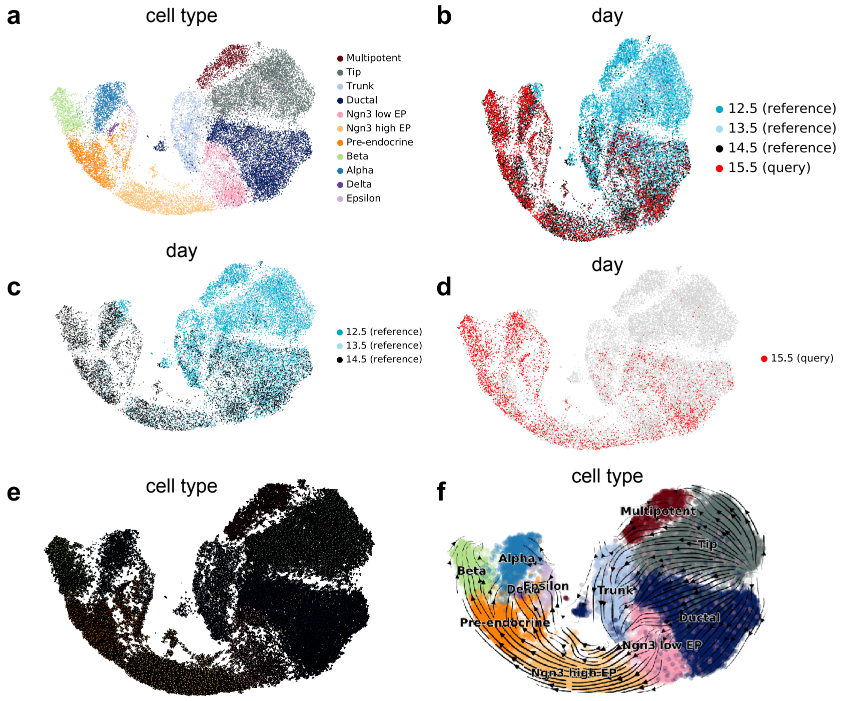

Supplementary Figure 13 | Reference mapping for Endocrine ($n = 22,163$) development. (a-d) UMAPs for latent representation of integrated reference and query data using scArches scANVI across cell types and time points. **(e-f)** Velocities derived using the whole data projected into a UMAP-based embedding at single-cell level (**e**) and stream plot (**f**).

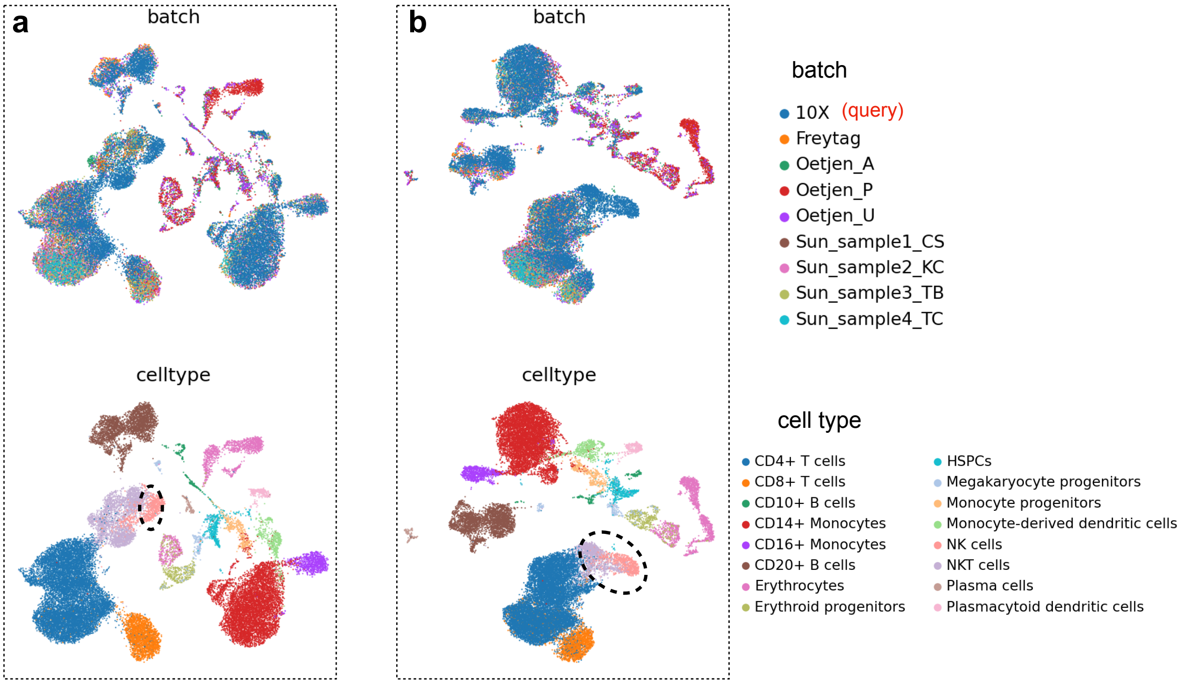

Supplementary Figure 14 | Reference mapping in presence of nuance cell types. (a-b) UMAPs for latent representation of integrated reference and query data using scArches scVI. The 10x batch is used as query while NK (**a**) or NK and NKT cells (**b**) are only present in the query data.

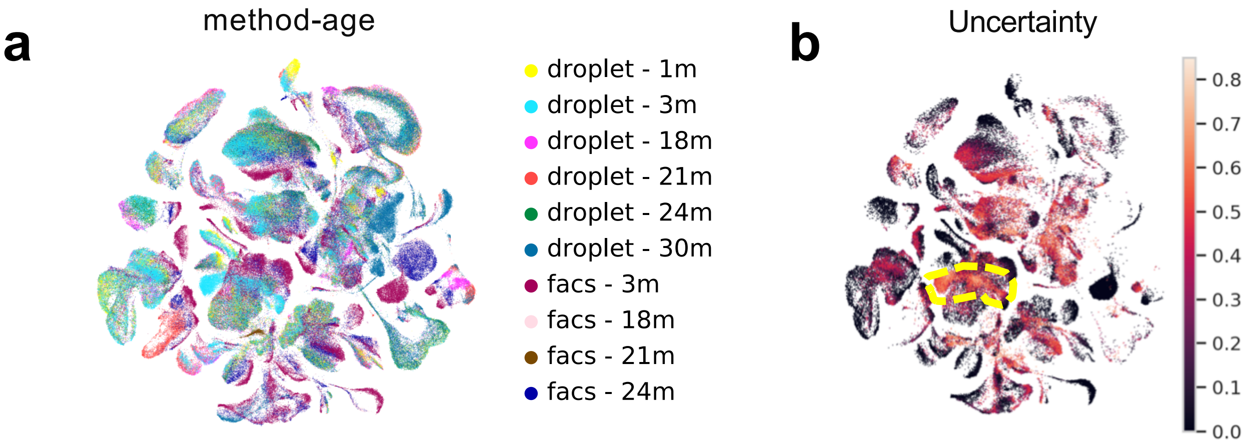

Supplementary Figure 15 | scArches successfully transfers knowledge from reference to query. (a) Querying Tabula Muris (n=90,120) to the larger reference atlas Tabula Senis (n=264,287) using scArches trVAE colored by different technologies and ages. **(b)** Reported uncertainty of the transferred labels. The highlighted tissue represents the trachea cells, which were removed from the reference data.

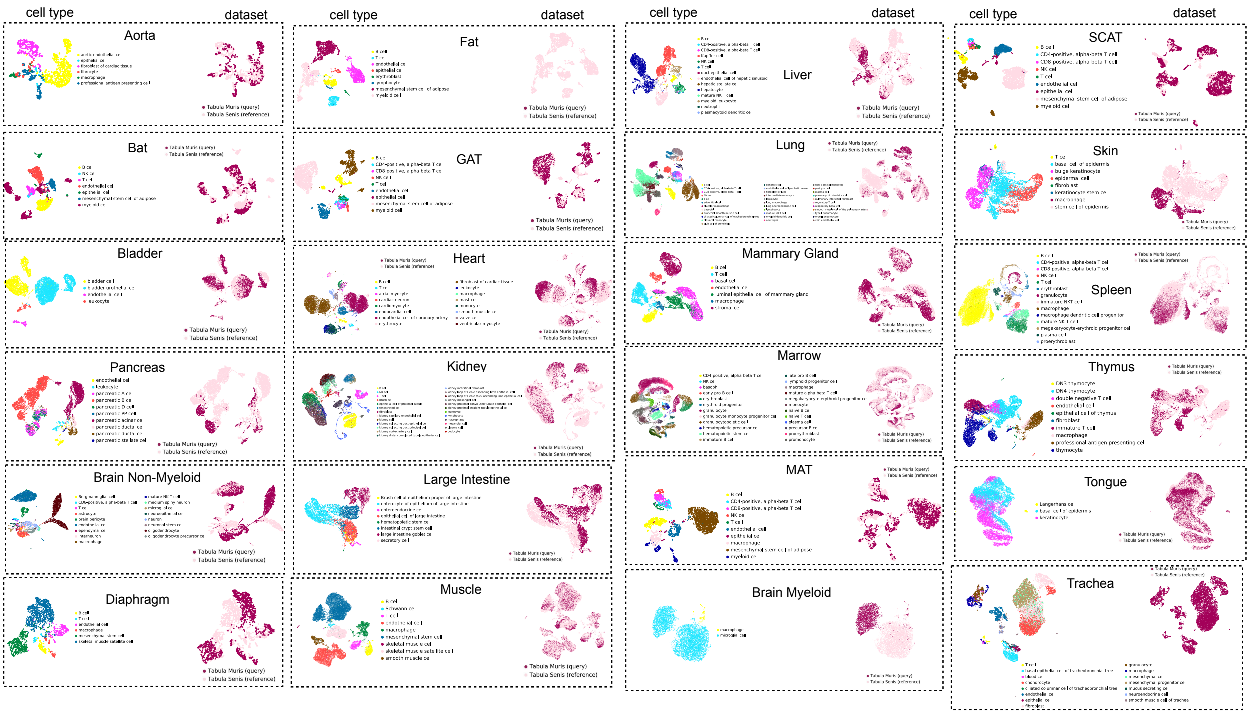

Supplementary Figure 16 | UMAPs for integrated TM and TS across 24 different tissues. UMAP plot of latent representation for integrated query TM and TS using scArches trVAE for different tissues colored by cell-types and the dataset.

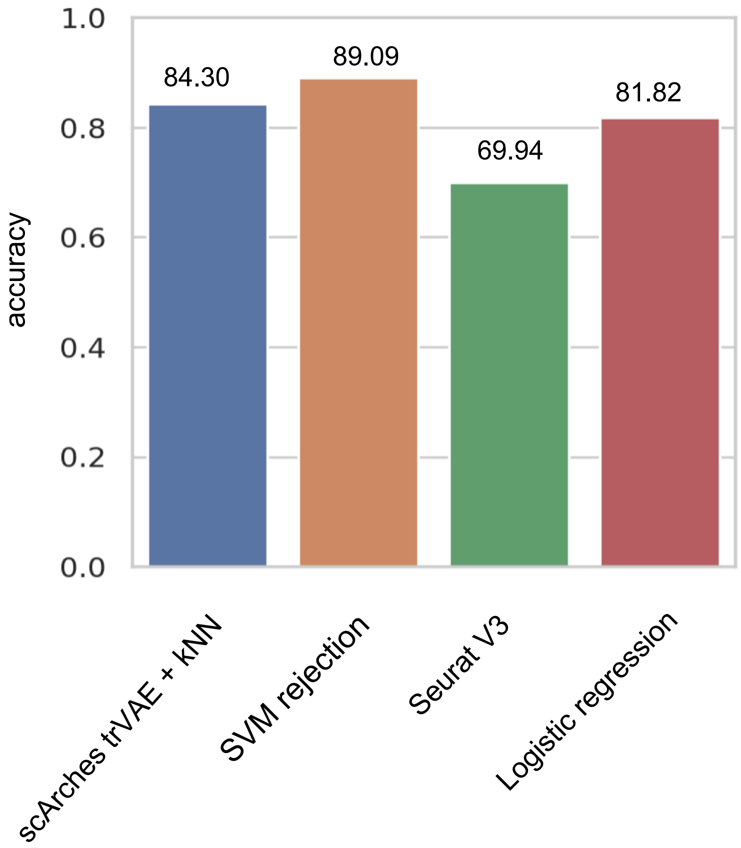

Supplementary Figure 17 | Comparison of scArches trVAE + kNN with existing automated cell type classification methods. Each classifier was trained on reference Tabula Senis data and the labels were predicted for query Tabula Muris. The Y axis denotes the accuracy (*#correct/#all_cells*) of classification for the query Tabula Muris data calculated for all cells excluding trachea.

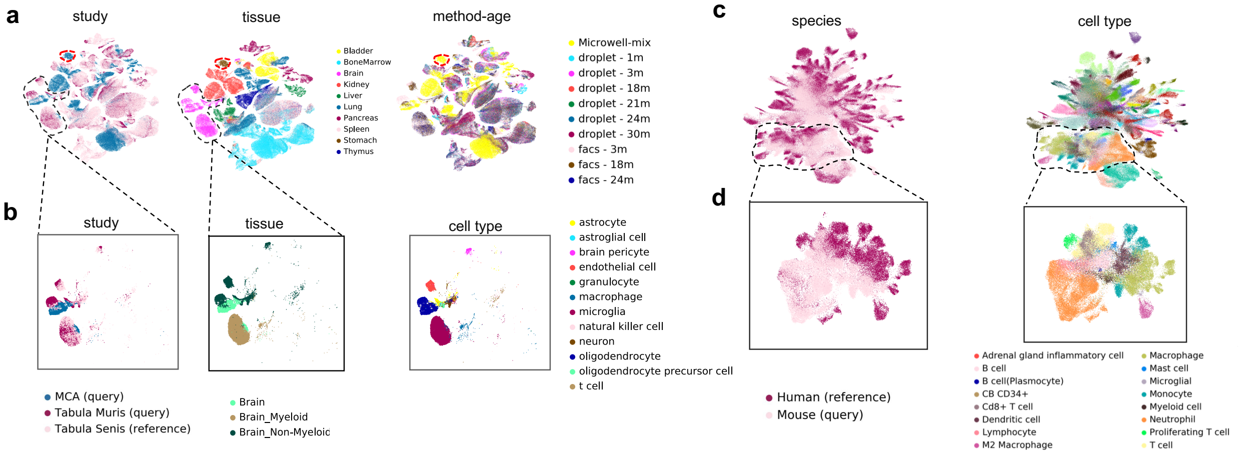

Supplementary Figure 18 | scArches trVAE successfully integrates data across whole organisms and between species. **(a)** Aligning query Mouse Cell Atlas (MCA, n=71, 259) and Tabula Muris (n=43, 127) mouse atlases into Tabula Senis (n=169, 425). The red dashed circle represents the stomach cells only present in MCA. **(b)** Alignment of query brain cells across both the myeloid and non-myeloid in the reference. **(c)** Querying MCA (n=122, 924) to the reference human cell atlas (n=249, 845). **(d)** The cross-species comparison between immune cells, illustrating mixedness across species. All the results were obtained using scArches trVAE base.

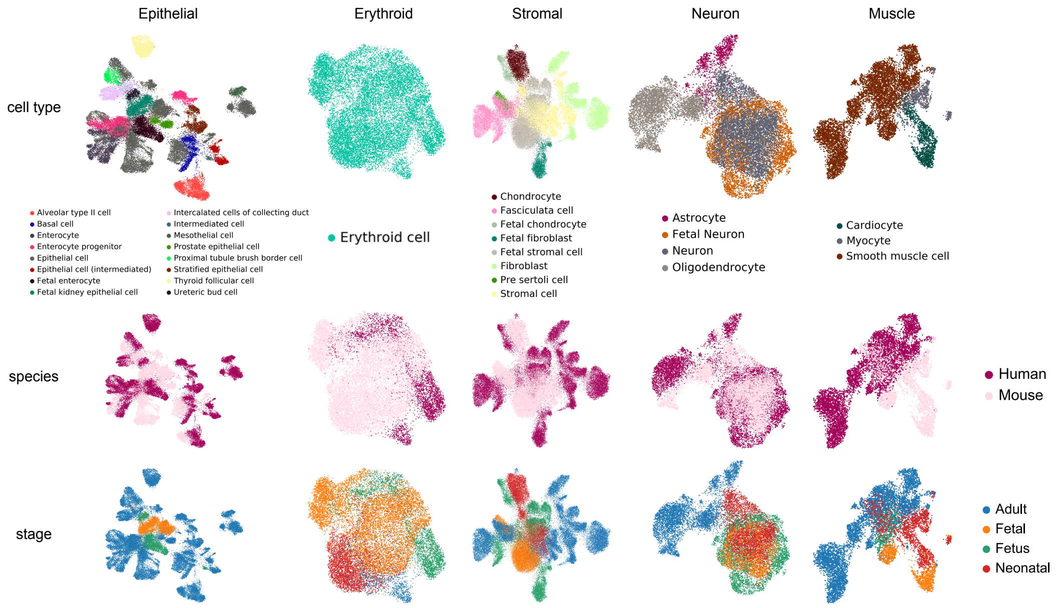

Supplementary Figure 19 | UMAP plots of major mammalian cell types across mouse and human. scArches trVAE integrated of query MCA (n=122, 924) and the reference human cell atlas (n=249, 845) across major mammalian cell types.

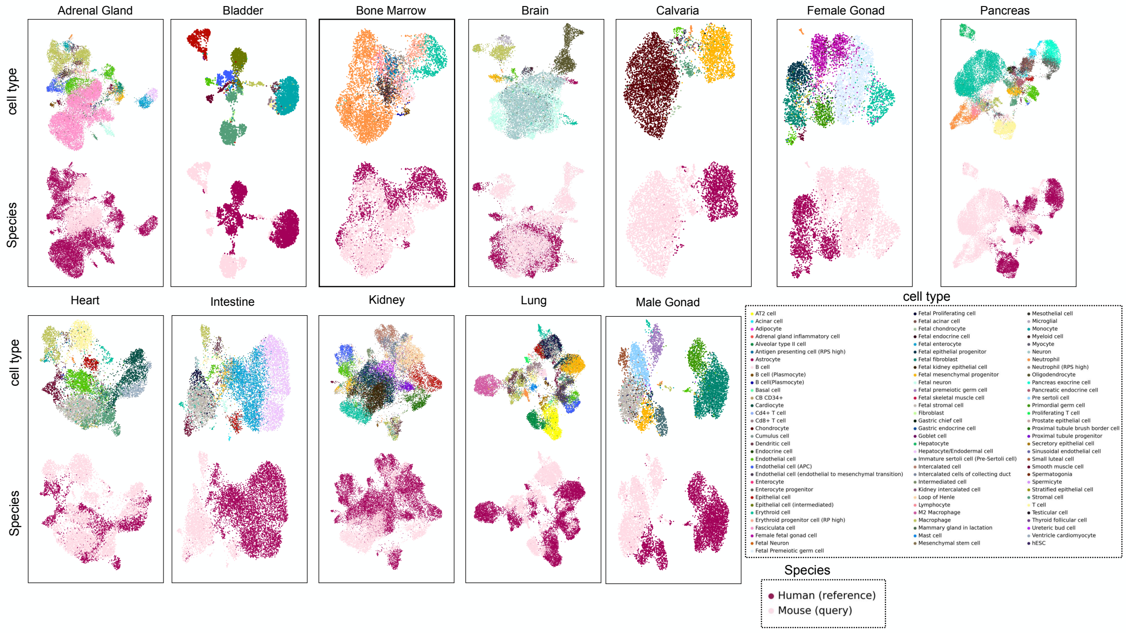

Supplementary Figure 20 | UMAP plots of shared tissues between mouse and human. scArches trVAE integrated of query MCA (n=122, 924) and the reference human cell atlas (n=249, 845) across different tissues.

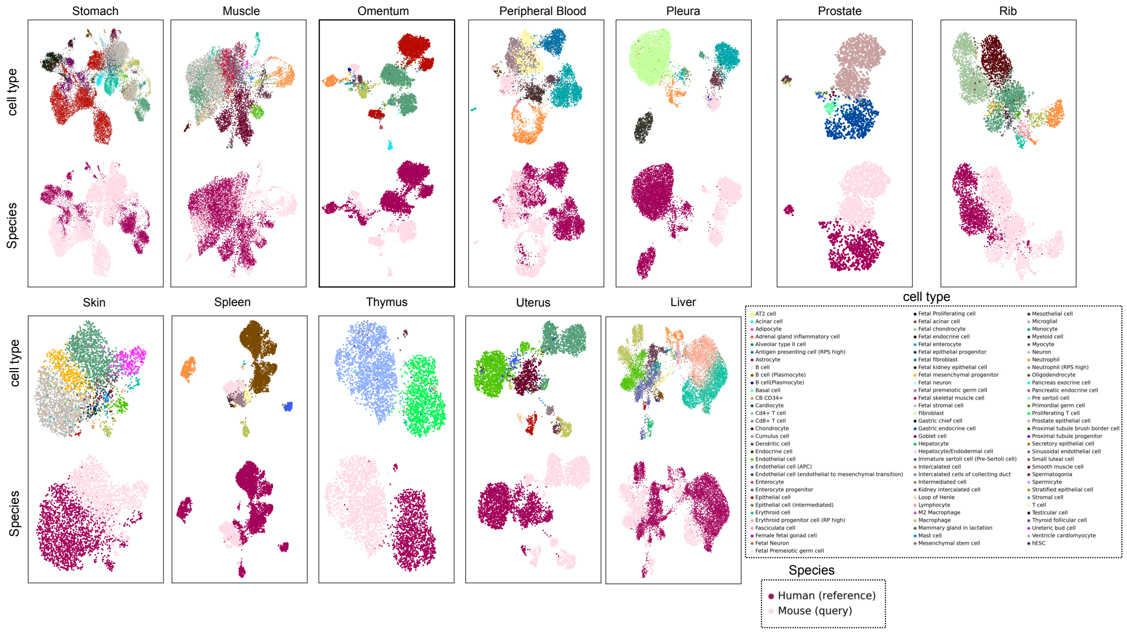

Supplementary Figure 21 | UMAP plots of shared tissues between mouse and human. scArches trVAE integrated of query MCA (n=122, 924) and the reference human cell atlas (n=249, 845) across different tissues.

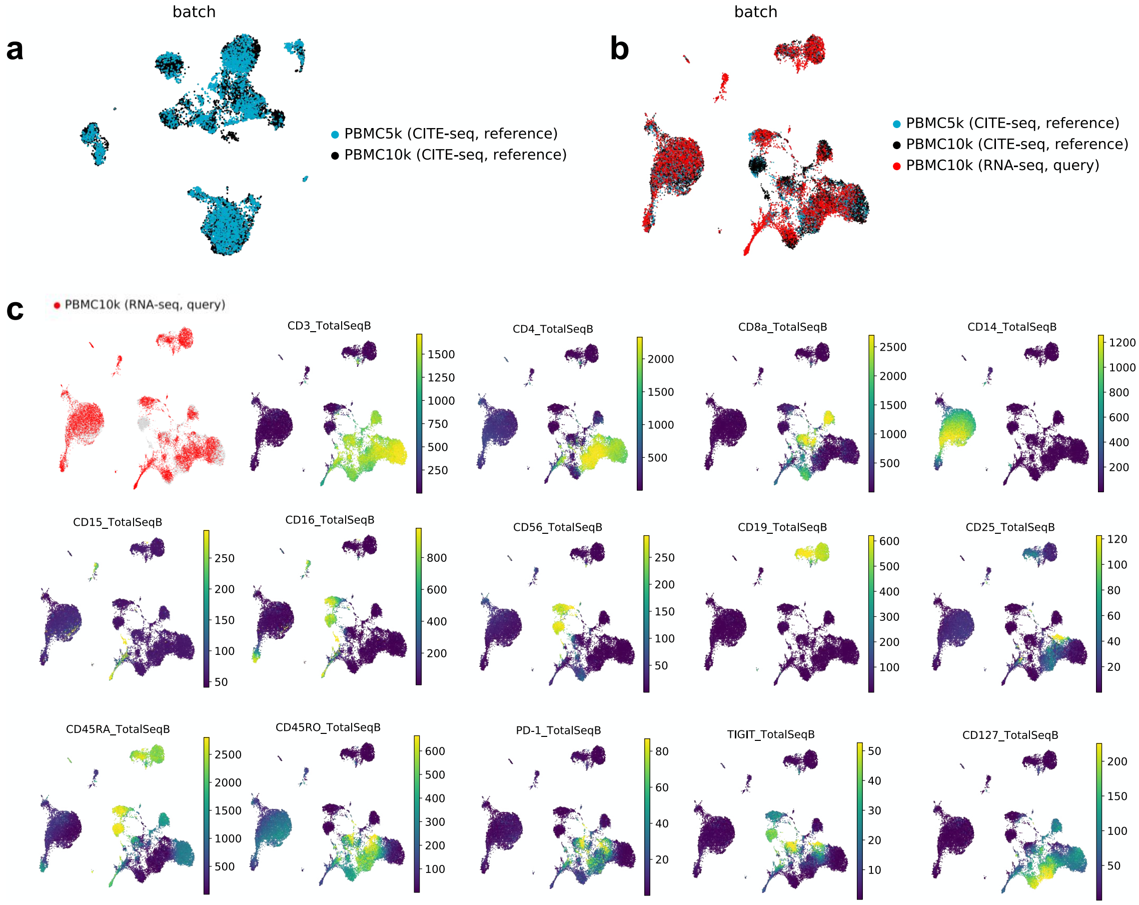

Supplementary Figure 22 | Multi-modal reference building and query mapping and imputation. **(a)** Integrated multi-modal CITE-seq reference using scArches totalVI. **(b)** Integration of query RNA-seq data in to the referees atlas. **(c)** Imputation of the missing protein data for query using reference proteins.

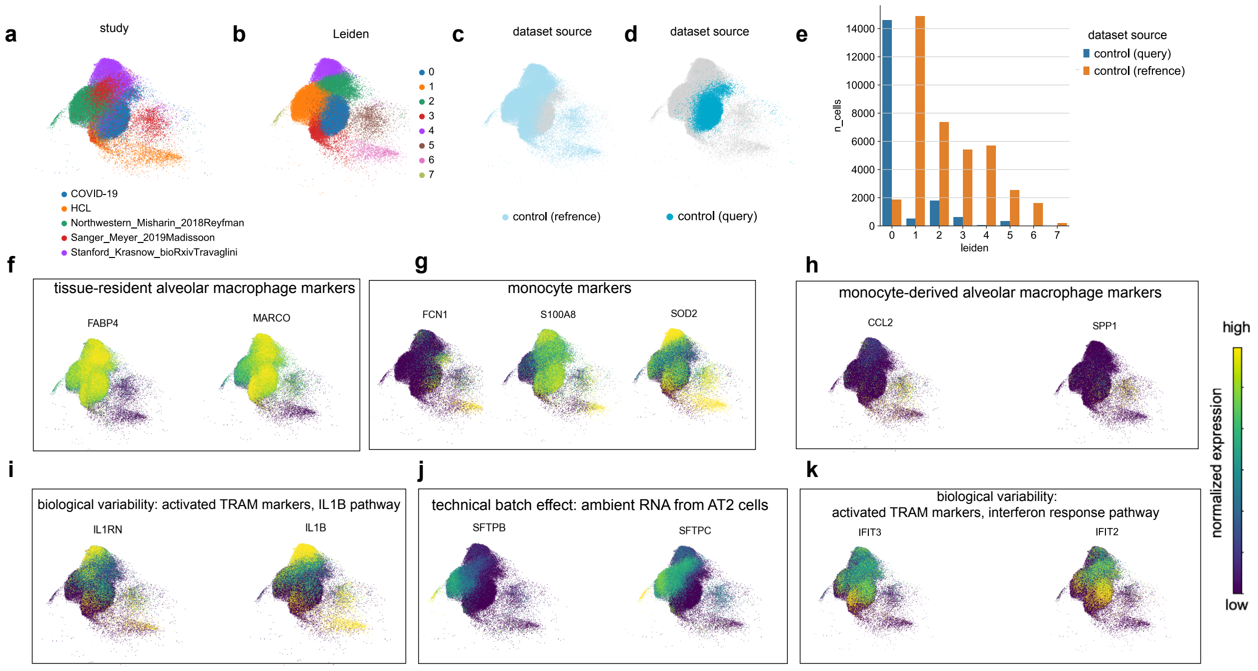

Supplementary Figure 23 | Extended analysis of biological variation for healthy macrophages in COVID-19 reference mapping. (a-d) UMAP of integrated control query and reference macrophages colored by datasets **(a)**, Leiden clustering **(b)**, and dataset source (**c-d**). **(e)** Composition of Leiden clusters colored by dataset source. **(f)** Comparison of various macrophage subpopulations. The results were obtained using scArches CVAE (NB).

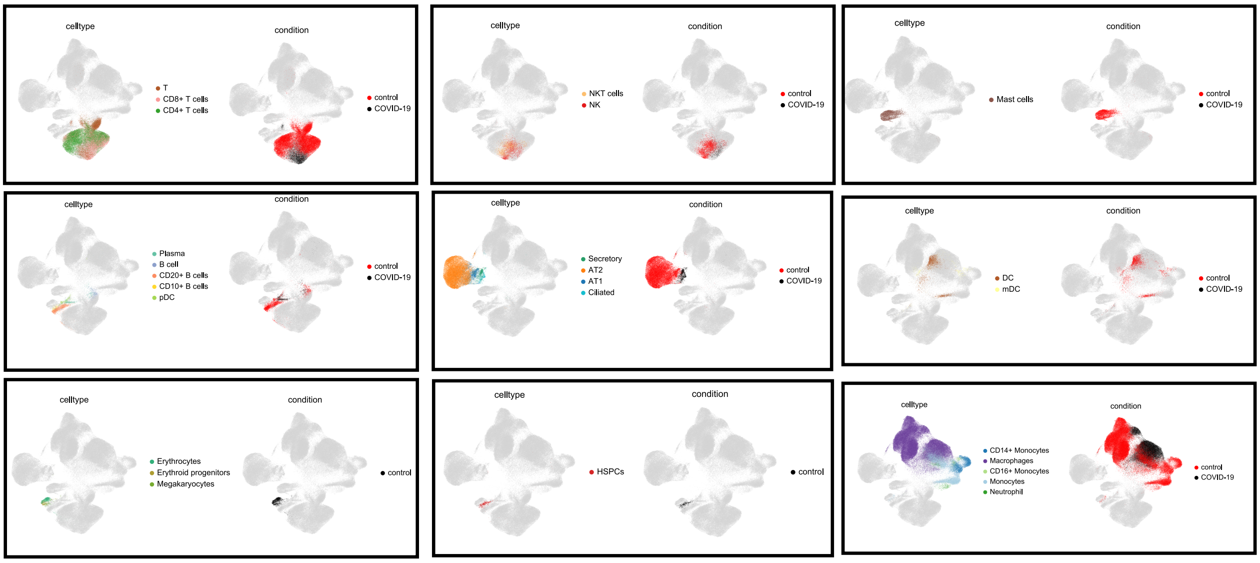

Supplementary Figure 24 | UMAP plots for all cell types present in query COVID-19 and reference. The plot denotes integrated and query data obtained using scArches CVAE (NB) colored cell-types and the condition.

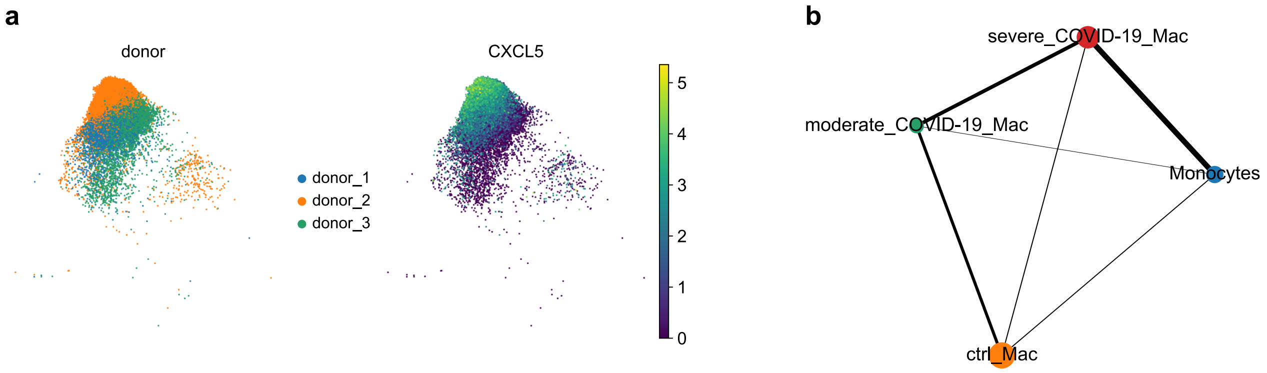

Supplementary Figure 25 | Relationship of monocyte-derived macrophages (MoMs) and monocytes in COVID-19 patients compared to a healthy reference. **(a)** cancer cells enriched with *CXLC5* from donor 2 in the Travaglini et al. dataset. **(b)** PAGA graph for monocyte and macrophage populations. Each node represents a cell state whose edge weights (represented as line thickness) quantify the connectivity between groups. The results were obtained using scArches CVAE (NB).

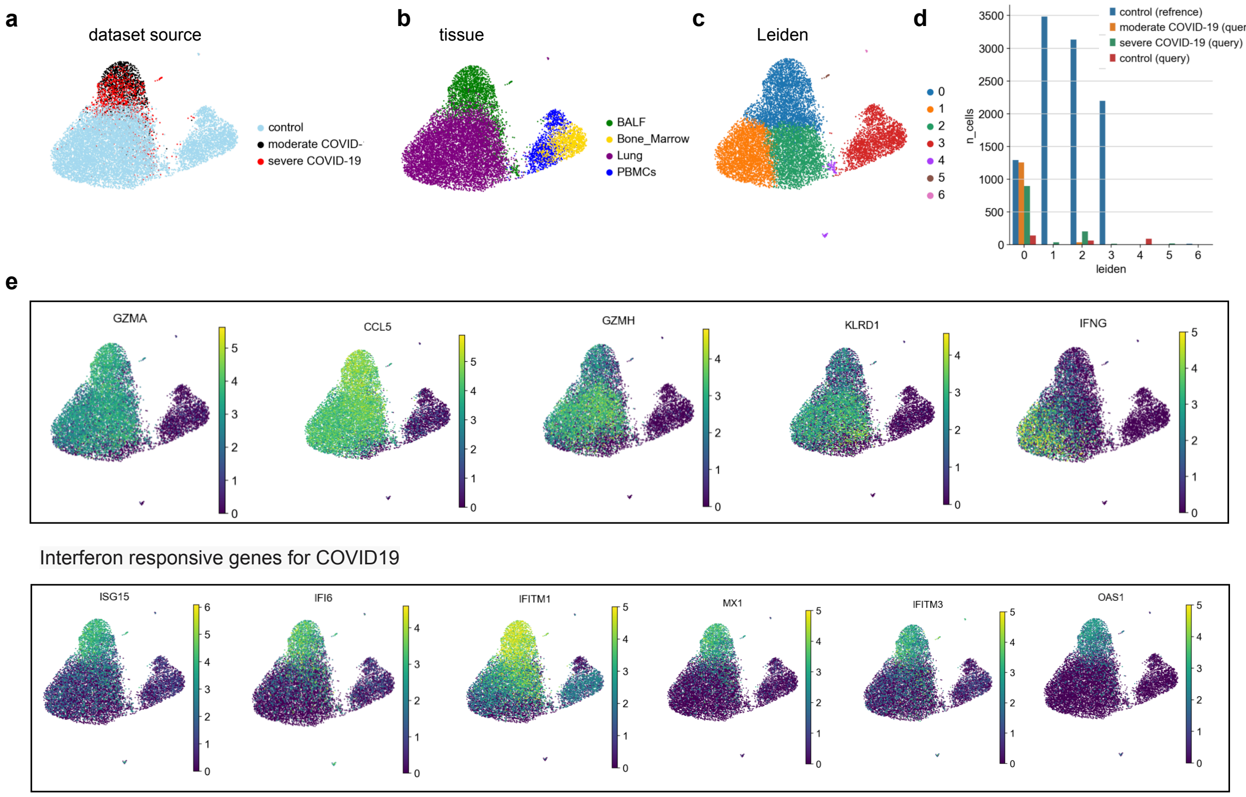

Supplementary Figure 26 | Extended analysis of CD8-T cells for COVID-19 reference mapping. **(a-b)** Separations of activated query CD8+ T cells from patients with COVID-19 from the rest. **(c-d)** UMAP of integrated reference and query CD8-T cells colored by Leiden cluster **(c)** and compositions of cells from each dataset sources in each cluster **(d)**. Markers for Lung and BALF CD8-T cells separating them from rest of tissues (**(e)**, first row). Expression of Interferon genes in COVID-19 query population (**(e)**, second row). The results were obtained using scArches CVAE (NB).

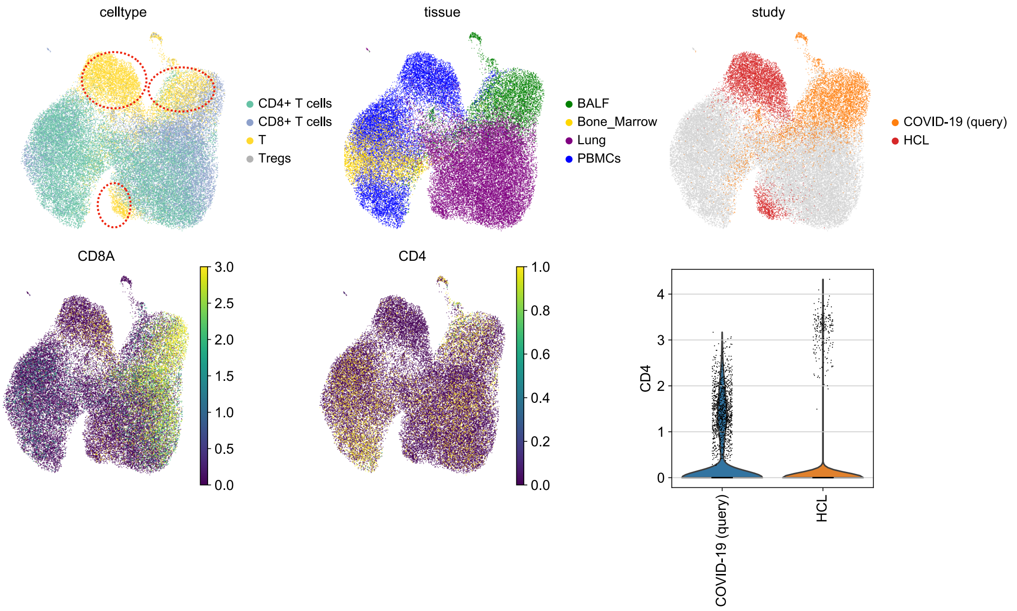

Supplementary Figure 27 | Overlap of T cell labels of all studies. Resolving T cell labels into CD8+ T cells, CD4+ T cells, and other T cells in HCL [1] and COVID-19 datasets. The highlighted populations were all annotated as T cells by original publications. The results were obtained using scArches CVAE (NB).

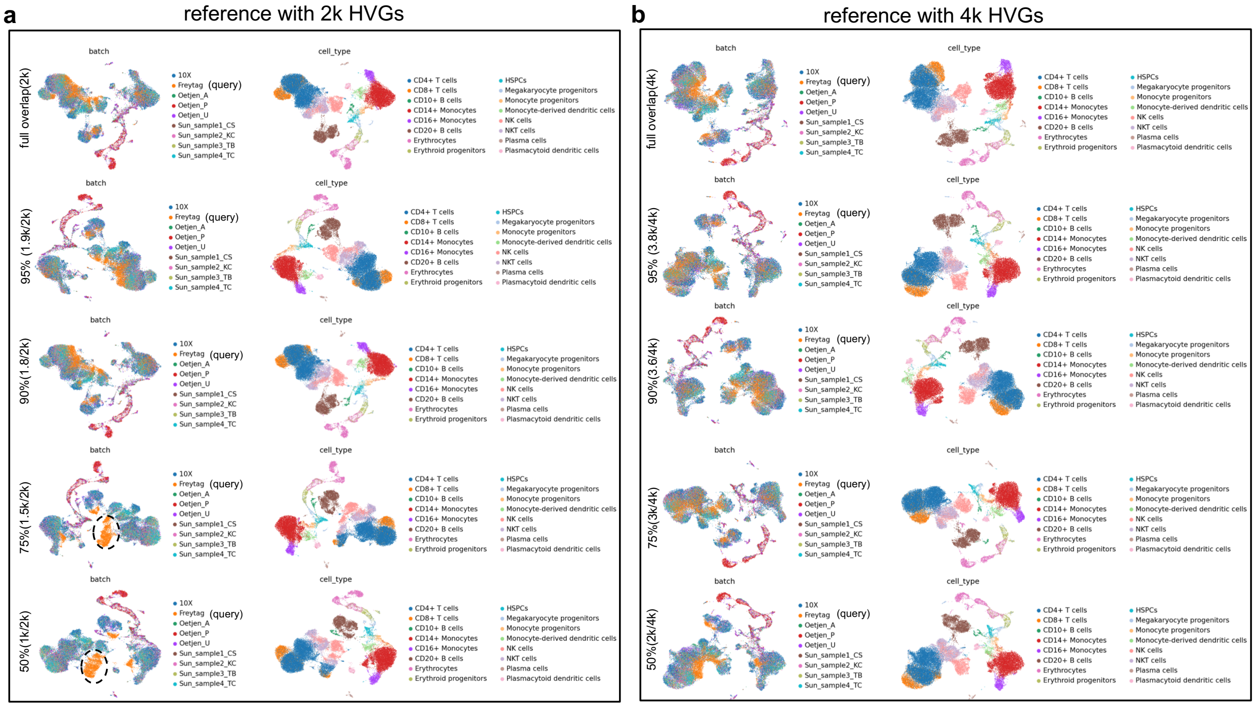

Supplementary Figure 28 | Assessing the effect for number of overlapping genes between query and reference on integration quality. (a-b) Training scArches scVI on Immune dataset with 2k (**a**) and 4k(**b**) HVGs while zero-filling the missing genes for query dataset. The percentage denotes number of shared genes between reference and query while the rest were missing in query data and filled with zeros to make it compatible with reference dataset. The highlighted populations represent major part of query data which failed to integrated in to reference data.
